# PDIA6–SCD1 Axis Rewires Lipid Metabolism to Drive Gastric Cancer Progression

**DOI:** 10.1002/advs.75923

**Published:** 2026-06-03

**Authors:** Zhen Tian, Yifan Cheng, Jiajie Zhou, Ruiqi Li, Shuai Zhao, Ben Li, Zijie Xu, Mengli Zi, Yayan Fu, Chenkai Zhang, Qiannan Sun, Shantanu Baral, Sen Wang, Daorong Wang

**Affiliations:** ^1^ Northern Jiangsu People's Hospital Clinical Teaching Hospital of Medical School Nanjing University Yangzhou China; ^2^ Northern Jiangsu People's Hospital Affiliated to Yangzhou University Yangzhou China; ^3^ Yangzhou Key Laboratory Of Basic And Clinical Transformation of Digestive And Metabolic Diseases Yangzhou China; ^4^ Department of General Surgery Northern Jiangsu People's Hospital Yangzhou China; ^5^ Department of General Surgery The First Affiliated Hospital of Nanjing Medical University Nanjing China

**Keywords:** cancer‐associated fibroblasts, gastric cancer, lipid metabolism, protein disulfide isomerase

## Abstract

Gastric cancer (GC) remains an aggressive malignancy with limited effective therapeutic options. Integrated single‐cell and bulk transcriptomic analyses identify protein disulfide isomerase A6 (PDIA6) as a tumor epithelial–enriched gene associated with advanced tumor‑ node‑metastasis (TNM) stage and unfavorable survival. Multi‐omics profiling reveals that PDIA6 drives lipid metabolic reprogramming by sustaining the monounsaturated fatty acid (MUFA)–enriched neutral lipid pools required for lipid droplet homeostasis and redox balance. Mechanistically, PDIA6 directly associates with stearoyl‐CoA desaturase 1 (SCD1) through a structure‐defined interface centered on Asp44 of SCD1, thereby restricting its ubiquitin–proteasome–mediated degradation and maintaining SCD1‐dependent fatty acid desaturation. In vivo, PDIA6 knockdown suppresses tumor growth and liver metastasis, and synergistic SCD1 inhibition (CAY10566) yields efficacy superior to monotherapies. Upstream, tumor–stromal interactions may contribute to PDIA6 upregulation, as cancer‐associated fibroblast‐derived C‐X‐C motif chemokine ligand 12 (CXCL12) activated C‐X‐C chemokine receptor 4 (CXCR4)‐dependent signal transducer and activator of transcription 3 (STAT3) signaling in vitro. These findings establish the PDIA6–SCD1 axis as a targetable lipid metabolic dependency in GC and position PDIA6 as a candidate therapeutic vulnerability for precision oncology.

## Graphical abstract

Protein disulfide isomerase A6 (PDIA6) is identified as an oncogenic driver in gastric cancer. PDIA6 directly binds and stabilizes SCD1 by limiting its ubiquitin–proteasome‐mediated degradation, thereby sustaining monounsaturated fatty acid (MUFA)‐enriched lipid homeostasis and lipid metabolic reprogramming. Co‑targeting PDIA6 and SCD1 synergistically suppresses tumor growth and liver metastasis, establishing the PDIA6–SCD1 axis as an actionable metabolic vulnerability.

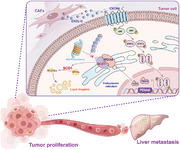

## 1 Introduction

Gastric cancer (GC) remains a major global health burden and a leading cause of cancer‐related mortality worldwide. In 2024, more than 968,000 new cases and approximately 660,000 deaths were reported worldwide [1]. Despite advances in surgery, chemotherapy, and targeted therapies, the prognosis of patients with advanced or metastatic GC remains poor. This is largely attributed to aggressive tumor growth, early dissemination, and limited therapeutic options [2, 3]. Therefore, elucidating the molecular mechanisms that drive GC progression is essential for identifying novel therapeutic targets.

The protein disulfide isomerase (PDI) family comprises endoplasmic reticulum (ER)‐resident chaperones that regulate protein folding, redox homeostasis, and quality control [4, 5, 6]. Increasing evidence has implicated this family in cancer progression [7, 8]. Among them, PDIA6 has emerged as a context‐dependent oncogenic regulator in several malignancies [9, 10, 11, 12]. Specifically, PDIA6 promotes pancreatic cancer progression and immune escape via CSN5‐mediated stabilization of β‐catenin and PD‐L1 [10]; attenuates cisplatin‐induced apoptosis and autophagy in non‐small cell lung cancer via MAP4K1/JNK/c‐Jun signaling [11]; enhances endometrial cancer cell proliferation and metastasis by modulating the TRPM2‐AS/miR‐424‐5p axis [9]; and supports nasopharyngeal carcinoma cell survival under therapeutic stress by preserving ER proteostasis [12]. However, its expression pattern, functional role, and mechanism of action in GC remain unclear.

Metabolic reprogramming is a hallmark of cancer that enables tumor cells to adapt to hostile microenvironmental conditions and sustain proliferative demand [13, 14, 15]. In GC, lipid metabolic reprogramming is particularly important, as lipids serve not only as structural components of membranes but also as regulators of signaling, energy homeostasis, and immune evasion [16, 17, 18]. Yet the molecular basis underlying lipid metabolic reprogramming in GC remains incompletely understood. Stearoyl‐CoA desaturase 1 (SCD1), the rate‐limiting enzyme for monounsaturated fatty acid synthesis, drives metabolic plasticity and tumor progression in multiple cancers [19, 20, 21, 22]. Here, our integrative proteomic and lipidomic analyses revealed a previously unrecognized link between PDIA6 and lipid metabolic homeostasis in GC, prompting us to investigate whether PDIA6 promotes GC progression through regulation of SCD1‐dependent lipid desaturation.

Beyond cell‐intrinsic regulatory mechanisms, PDIA6 expression may also be modulated by cues from the tumor microenvironment (TME). Signal transducer and activator of transcription 3 (STAT3) is a master oncogenic transcription factor that integrates proliferative, survival, and inflammatory signaling cascades in multiple malignancies [23, 24]. Notably, STAT3 activity is frequently activated by TME‐derived paracrine signals, with cancer‐associated fibroblasts (CAFs) representing the most abundant and functionally dominant stromal cell type [25, 26, 27]. Among the CAF‐derived bioactive mediators, C‐X‐C motif chemokine ligand 12 (CXCL12) is a well‐characterized ligand that activates CXCR4‐dependent JAK‐STAT3 signaling and has recently emerged as a critical regulator of metabolic reprogramming within the TME [28, 29, 30]. However, whether the CAF‐CXCL12‐STAT3 axis regulates PDIA6 expression in GC remains unknown.

In the present study, integrative multi‐omics analyses combined with clinical validation identify PDIA6 as an aberrantly upregulated gene in GC that is associated with poor prognosis. Functionally, PDIA6 promotes GC cell proliferation, migratory capacity, and intracellular lipid accumulation. Mechanistically, PDIA6 directly interacts with SCD1 and limits its ubiquitin‐proteasome‐mediated degradation, thereby sustaining fatty acid desaturation and lipid droplet homeostasis. Tumor‐stromal signaling may also contribute to PDIA6 upregulation, providing an upstream regulatory context. Together, these findings define a PDIA6‐SCD1 regulatory axis linking protein quality control to lipid metabolic reprogramming and nominate PDIA6 as a potential therapeutic vulnerability in GC.

## 2 Results

### 2.1 Identification of PDIA6 as an Oncogenic Driver in GC

**FIGURE 1:**
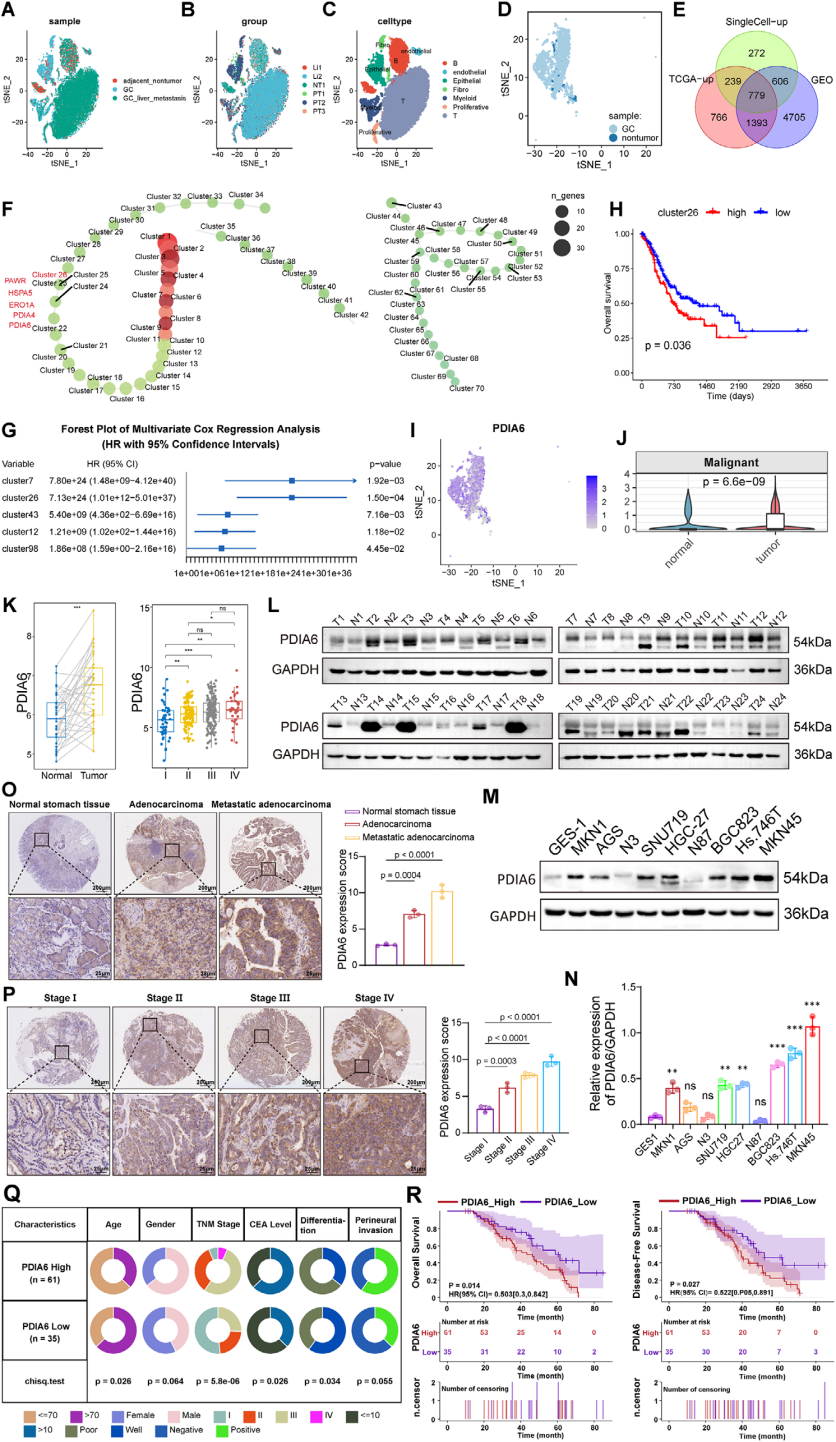
Identification of PDIA6 as a tumor‐enriched prognostic marker in GC. (A–C) t‐SNE visualization of single‐cell transcriptomes from GSE163558, annotated by tissue origin, patient group, and major cell types. (D) Subset t‐SNE analysis of epithelial cells showing transcriptional divergence between tumor and adjacent non‐tumor epithelium. (E) Venn diagram of overlapping genes upregulated in tumor epithelial cells from GSE163558 and bulk RNA‐seq datasets (TCGA‐STAD and GSE56807; |log_2_ fold change| > 2, p < 0.05). (F) Markov clustering (MCL) of the 779 intersecting genes. G) Forest plot of multivariate Cox regression analysis of ssGSEA‐based cluster enrichment scores for overall survival (GSE84426 cohort). (H) Kaplan–Meier analysis of overall survival in TCGA‐STAD patients stratified by cluster26 enrichment. (I,J) Single‐cell feature and violin plots comparing PDIA6 expression between tumor‐associated and adjacent non‐tumor epithelial cells. K) TCGA‐STAD analysis of PDIA6 expression in GC tissues and across TNM stages. (L) Western blot analysis of PDIA6 protein levels in 24 paired GC tissues (T) and matched adjacent non‐tumor tissues (N). (M,N) Western blot analysis and densitometric quantification of PDIA6 protein levels in GC cell lines relative to GES‐1 cells. (O,P) Representative immunohistochemical (IHC) staining and quantitative analysis of PDIA6 in normal gastric tissues, non‐metastatic and metastatic gastric adenocarcinomas, and across AJCC stages I–IV. (Q) Clinicopathological characteristics of 96 GC patients stratified by PDIA6 expression based on IHC scores. (R) Kaplan–Meier analysis of overall and disease‐free survival in the same cohort. Data are presented as mean ± SD. *p < 0.05, **p < 0.01, ***p < 0.001; ns, not significant.

Single‐cell RNA sequencing (scRNA‐seq) data from primary GC, GC liver metastases, and matched adjacent non‐tumor tissues (GSE163558) were analyzed. After quality control and filtering, t‐distributed stochastic neighbor embedding (t‐SNE) resolved the major cellular populations across the samples (Figure 1A–C; Figure S1A). Tumor‐associated epithelial cells were extracted for focused analysis, and the t‐SNE subset revealed marked transcriptional heterogeneity between the tumor and adjacent non‐tumor epithelial cells (Figure 1D). Differential expression analysis identified significantly upregulated genes in tumor epithelial cells (|log_2_FC| > 2, p < 0.05). Integration of these genes with the transcripts upregulated in The Cancer Genome Atlas‐Stomach Adenocarcinoma (TCGA‐STAD) and GSE56807 bulk RNA‐seq datasets yielded 779 consistently upregulated genes (Figure 1E). Markov Cluster Algorithm (MCL)–based co‐expression analysis partitioned the 779 genes into distinct clusters (Figure 1F). Single‐sample gene set enrichment analysis (ssGSEA) followed by multivariate Cox regression in the GSE84426 cohort identified five survival‐associated clusters (Figure 1G), among which cluster26 was independently validated in TCGA‐STAD (Figure 1H; Figure S1B; Supplementary file S1). Within cluster26, PDIA6 showed the strongest negative association with overall survival in both cohorts (Figure S1C,D). At single‐cell resolution, PDIA6 expression was predominantly restricted to malignant epithelial cells, with low expression in stromal and immune populations (Figure 1I,J; Figure S1E). Consistently, TCGA‐STAD analysis showed that PDIA6 was substantially elevated in GC tissues relative to adjacent non‐tumor tissues and progressively increased with the tumor‐node‐metastasis (TNM) stage (Figure 1K). Based on its tumor‐enriched expression and prognostic relevance, PDIA6 was selected for further analyses.

To substantiate its clinical relevance, PDIA6 expression was examined in GC specimens and cell lines. Western blot analysis confirmed higher PDIA6 protein levels in GC tissues than in matched adjacent non‐tumor tissues (Figure 1L) and consistently elevated expression across multiple GC cell lines relative to GES‐1 cells (Figure 1 M,N). Immunohistochemistry (IHC) staining further showed stepwise upregulation from non‐tumor to non‐metastatic and metastatic gastric adenocarcinomas, with higher levels observed in advanced TNM stages (Figure 1O,P). Clinicopathological correlation analysis revealed that high PDIA6 expression was associated with poor tumor differentiation, perineural invasion, elevated serum carcinoembryonic antigen, and advanced TNM stage (Figure 1Q). Cox regression analysis identified the PDIA6 IHC score as an independent prognostic factor for GC, and Kaplan–Meier analysis showed that high PDIA6 expression was correlated with substantially shorter overall and disease‐free survival (Figure 1R; Tables S1 and S2).

### 2.2 PDIA6 Promotes GC Proliferation and Migration in Vitro and in Vivo

**FIGURE 2:**
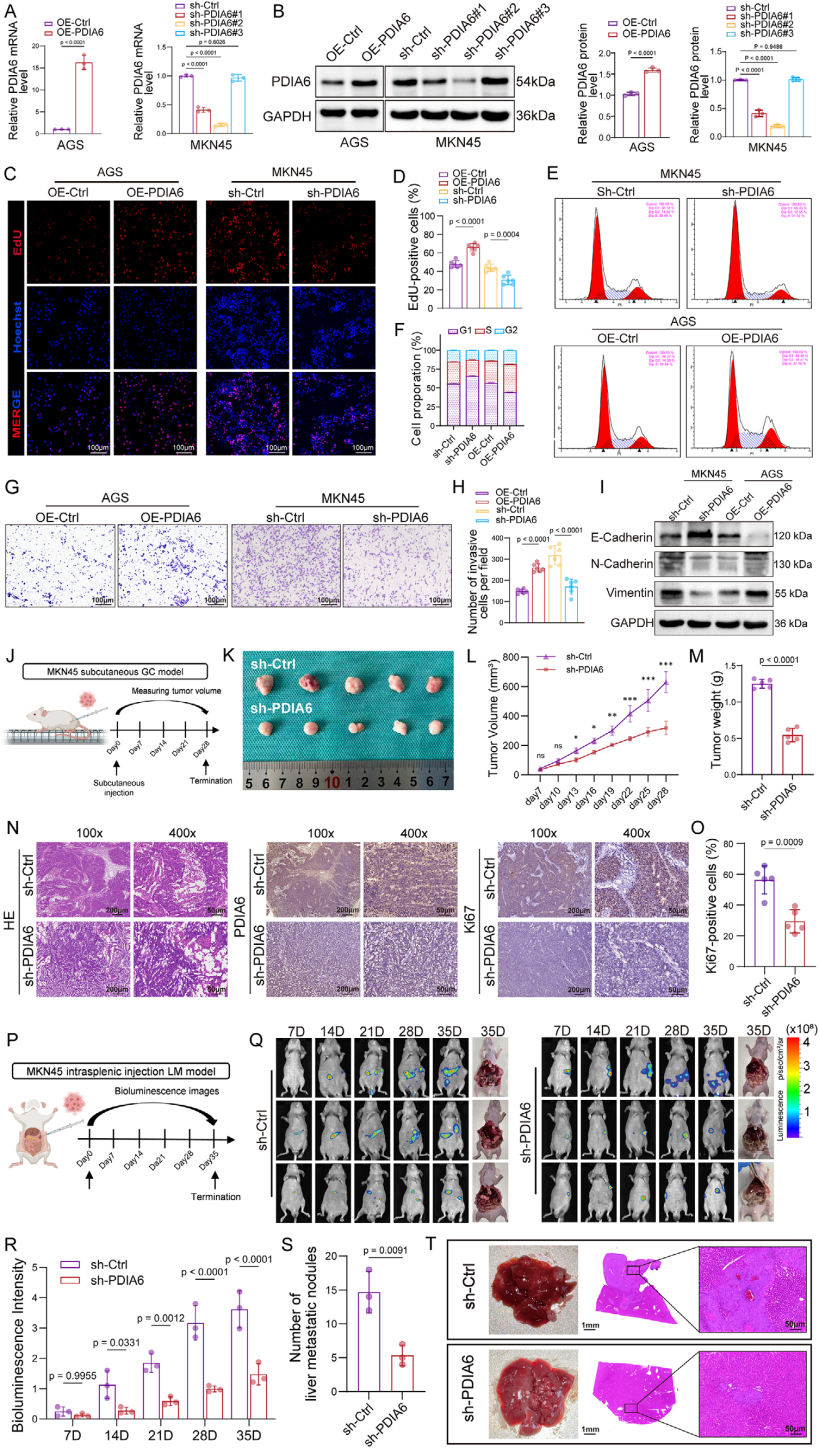
PDIA6 promotes GC cell proliferation and migration in vitro and in vivo. (A,B) qRT‐PCR and western blot analyses of PDIA6 mRNA and protein levels. (C,D) Representative images and quantification of EdU incorporation assays. (E,F) Flow cytometric analysis of cell‐cycle distribution and corresponding quantification of the percentages of cells in G1, S, and G2 phases. (G,H) Representative images and quantification of Transwell migration assays. (I) Western blot analysis of epithelial–mesenchymal transition (EMT)‐related markers. (J) Schematic illustration of the subcutaneous xenograft model. (K) Representative images of xenograft tumors derived from control or PDIA6‐knockdown MKN45 cells in BALB/c nude mice at the study endpoint (n = 5 mice per group). L) Growth curves of xenograft tumors. (M) Quantification of xenograft weights. (N) Representative hematoxylin and eosin (H&E) images and IHC staining of Ki67 and PDIA6 in xenograft tissues. (O) Quantification of Ki67‐positive cells. (P) Schematic illustration of the splenic injection liver metastasis model. (Q–T) Fluorescence imaging, gross liver images, quantification of metastatic burden, and H&E staining of liver metastatic lesions derived from control or PDIA6‐knockdown MKN45 cells (n = 3 mice per group). For in vitro assays shown in A–I, experiments were performed in PDIA6‐knockdown MKN45 cells and PDIA6‐overexpressing AGS cells. Data are presented as mean ± SD. *, *p* < 0.05; **, *p* < 0.01; ***, *p* < 0.001; ns, not significant.

Given its elevated expression in GC, the functional role of PDIA6 in tumor cell proliferation and migration was examined. AGS cells with relatively low endogenous PDIA6 expression were used for gain‐of‐function assays, whereas MKN45 cells with relatively high basal PDIA6 expression were used for loss‐of‐function assays. Among the three independent short hairpin RNAs, shPDIA6#2 showed the highest silencing efficiency and was therefore selected for subsequent experiments. PDIA6 modulation was confirmed at both the mRNA and protein levels using quantitative reverse transcription (qRT)‐polymerase chain reaction (PCR) and western blotting (Figure 2A,B). The 5‐ethynyl‐2′‐deoxyuridine (EdU) incorporation assays showed that PDIA6 knockdown markedly reduced the proportion of EdU‐positive nuclei in MKN45 cells, whereas PDIA6 overexpression substantially increased EdU incorporation into AGS cells (Figure 2C,D). Cell‐cycle profiling revealed that PDIA6 knockdown induced G1 accumulation with reduced S‐phase cells, whereas PDIA6 overexpression increased the S and G2 phases (Figure 2E,F). Transwell migration assays demonstrated that PDIA6 knockdown impaired the migratory capacity of MKN45 cells, and PDIA6 overexpression enhanced the migration of AGS cells (Figure 2G,H). Analysis of epithelial–mesenchymal transition (EMT) markers showed that PDIA6 knockdown increased E‐cadherin expression and decreased N‐cadherin and vimentin expression, whereas the opposite pattern was observed upon PDIA6 overexpression (Figure 2I), thus supporting a pro‐migratory role.

To validate these observations in vivo, we established subcutaneous xenograft and splenic injection‐based liver metastasis models. In the subcutaneous xenograft model, PDIA6 knockdown markedly suppressed tumor growth in BALB/c nude mice (n = 5 mice per group), as evidenced by the reduced tumor size, slower growth kinetics, and lower tumor weight at the endpoint (Figure 2J–M). Ki67 staining was consistently decreased in PDIA6‐knockdown tumors relative to controls (Figure 2N,O). In the liver metastasis model, PDIA6 knockdown substantially reduced hepatic colonization five weeks after splenic injection (n = 3 mice per group), as indicated by lower fluorescence intensity, reduced gross metastatic burden, and fewer histologically confirmed metastatic lesions (Figure 2P–T). Collectively, these results indicate that PDIA6 promotes GC cell proliferation, migration, and metastatic dissemination both in vitro and in vivo, thereby supporting its pro‐tumorigenic role in GC progression.

### 2.3 PDIA6 Drives Lipid Metabolic Reprogramming in GC

**FIGURE 3:**
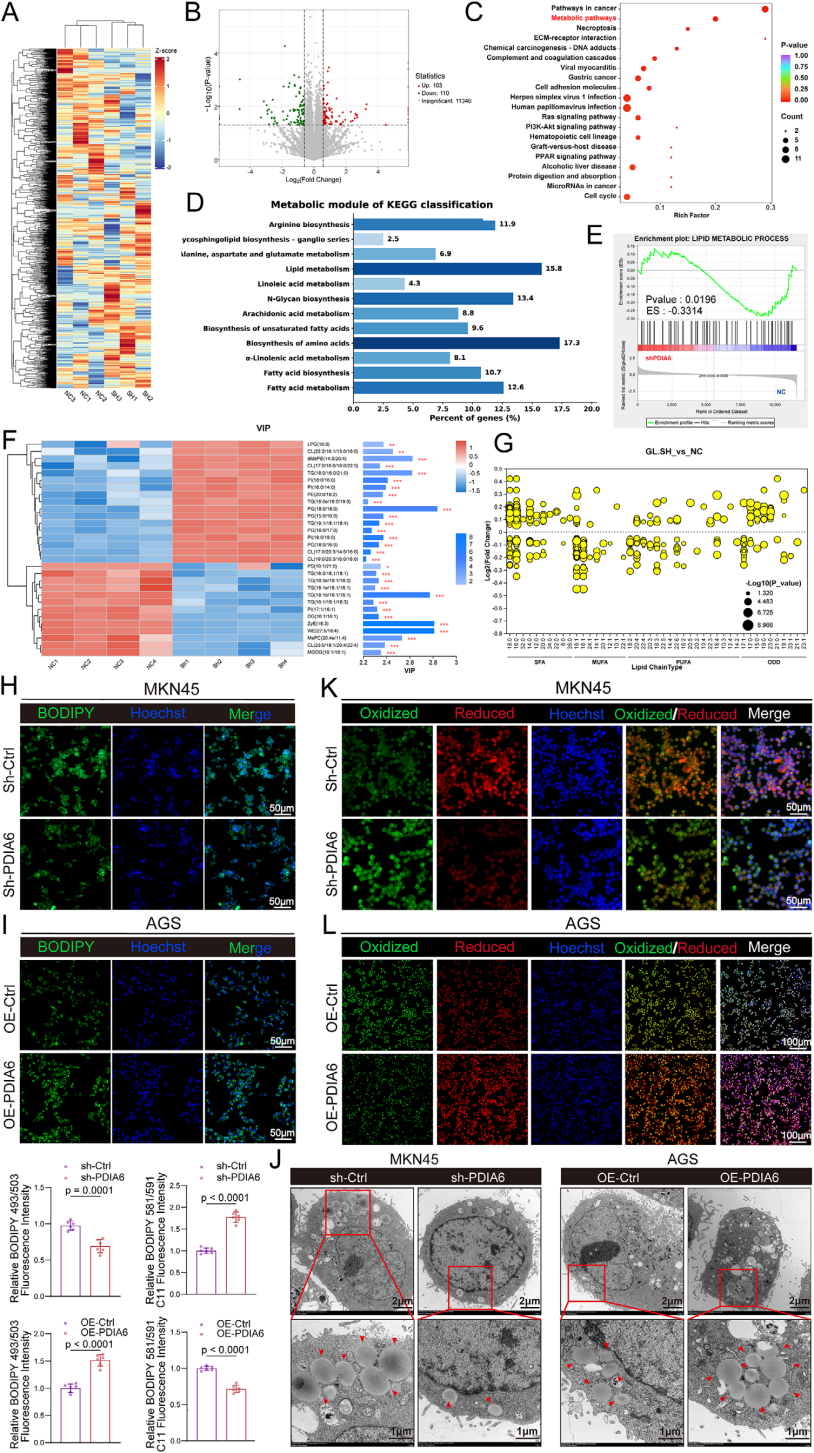
PDIA6 drives lipid metabolic reprogramming in GC. (A) Heatmap of differentially expressed proteins (DEPs) identified by DIA‐based quantitative proteomic analysis. (B) Volcano plot of DEPs between control and PDIA6‐knockdown MKN45 cells. (C) Bubble plot of Kyoto Encyclopedia of Genes and Genomes (KEGG) pathway enrichment analysis of DEPs. (D) Bar plot of KEGG‐classified metabolic pathways enriched among DEPs. (E) Gene set enrichment analysis (GSEA) plot showing downregulation of lipid metabolic processes after PDIA6 knockdown. (F) Hierarchical clustering heatmap and variable importance in projection (VIP) analysis of the top 30 differentially abundant lipid species in control and PDIA6‐knockdown MKN45 cells. The right panel shows the VIP values of the corresponding lipid species, with bar colors indicating statistical significance based on Student's t‐test. *, *p* < 0.05; **, *p* < 0.01; and ***, *p* < 0.001. (G) Bubble plot showing differential lipid acyl‐chain unsaturation patterns between control and PDIA6‐knockdown MKN45 cells. The x‐axis indicates acyl‐chain types, the y‐axis represents log2(fold change), and each bubble denotes an individual lipid species. (H,I) BODIPY 493/503 staining and quantification of neutral lipid accumulation in PDIA6‐knockdown MKN45 cells and PDIA6‐overexpressing AGS cells. J) Transmission electron microscopy of lipid droplets in PDIA6‐knockdown MKN45 cells and PDIA6‐overexpressing AGS cells. (K,L) BODIPY 581/591 C11 staining and quantification of lipid peroxidation in PDIA6‐knockdown MKN45 cells and PDIA6‐overexpressing AGS cells. Data are presented as mean ± SD and statistical significance is indicated.

To investigate the molecular basis of PDIA6‐mediated phenotypes in GC cells, ultra‐fast ultrafast data‐independent acquisition (DIA)‐based quantitative proteomic profiling was performed in control and PDIA6‐knockdown MKN45 cells (Figure 3A; Figure S2A–D). Differential expression analysis revealed 213 differentially expressed proteins (DEPs; |log_2_FC| ≥ 1.5 or ≤ 0.67, p < 0.05; Figure 3B). Kyoto Encyclopedia of Genes and Genomes (KEGG) pathway enrichment analysis revealed that metabolic pathways were among the most prominently altered categories, with lipid metabolism emerging as a highly enriched submodule (Figure 3C,D). Consistently, gene set enrichment analysis (GSEA) revealed significant downregulation of lipid metabolic processes following PDIA6 knockdown (ES = −0.3314, p = 0.0196; Figure 3E).

To further define these metabolic changes, untargeted liquid chromatography (LC)‐tandem mass spectrometry (MS/MS) lipidomic profiling was performed in the control and PDIA6‐knockdown MKN45 cells (Figure S3A–E). Heatmap visualization and variable importance in projection (VIP) analysis of the top 30 differentially abundant lipid species revealed high within‐group consistency and clear separation between control and PDIA6‐knockdown MKN45 cells, indicating marked lipidomic remodeling following PDIA6 knockdown (Figure 3F). Bubble plot analysis of differential lipid acyl‐chain composition further revealed a shift toward a less desaturated lipid landscape, characterized by a widespread decrease in lipid species containing the major monounsaturated fatty acyl chains (C16:1 and C18:1), together with a relative increase in species enriched in saturated acyl chains, particularly C16:0 and C18:0 (Figure 3G). Consistent with this pattern, multiple monounsaturated fatty acid (MUFA)‐containing triacylglycerol (TG) and diacylglycerol (DG) species were reduced, whereas several more saturated phospholipid species, including phosphatidylinositol (PI, 18:0/16:0) and phosphatidylglycerol (PG, 18:0/18:0), were elevated in the PDIA6‐knockdown cells (Figure 3G; Figure S3F–J). Together, these findings indicate that PDIA6 helps preserve a MUFA‐enriched lipid state and neutral lipid homeostasis in GC cells.

These lipidomic alterations were further confirmed using functional assays. BODIPY 493/503 staining revealed that PDIA6 knockdown decreased neutral lipid accumulation in MKN45 cells, whereas PDIA6 overexpression increased neutral lipid accumulation in AGS cells (Figure 3H,I). Consistently, transmission electron microscopy revealed fewer and smaller lipid droplets after PDIA6 knockdown, but more abundant and enlarged ones upon PDIA6 overexpression (Figure 3J). Lipid peroxidation, as assessed by BODIPY 581/591 C11 staining, was increased by PDIA6 knockdown in MKN45 cells but reduced by PDIA6 overexpression in AGS cells (Figure 3K,L). Collectively, these results indicate that PDIA6 drives lipid metabolic reprogramming in GC by sustaining lipid storage, lipid droplet homeostasis, and redox balance.

### 2.4 PDIA6 Physically Interacts With SCD1

**FIGURE 4:**
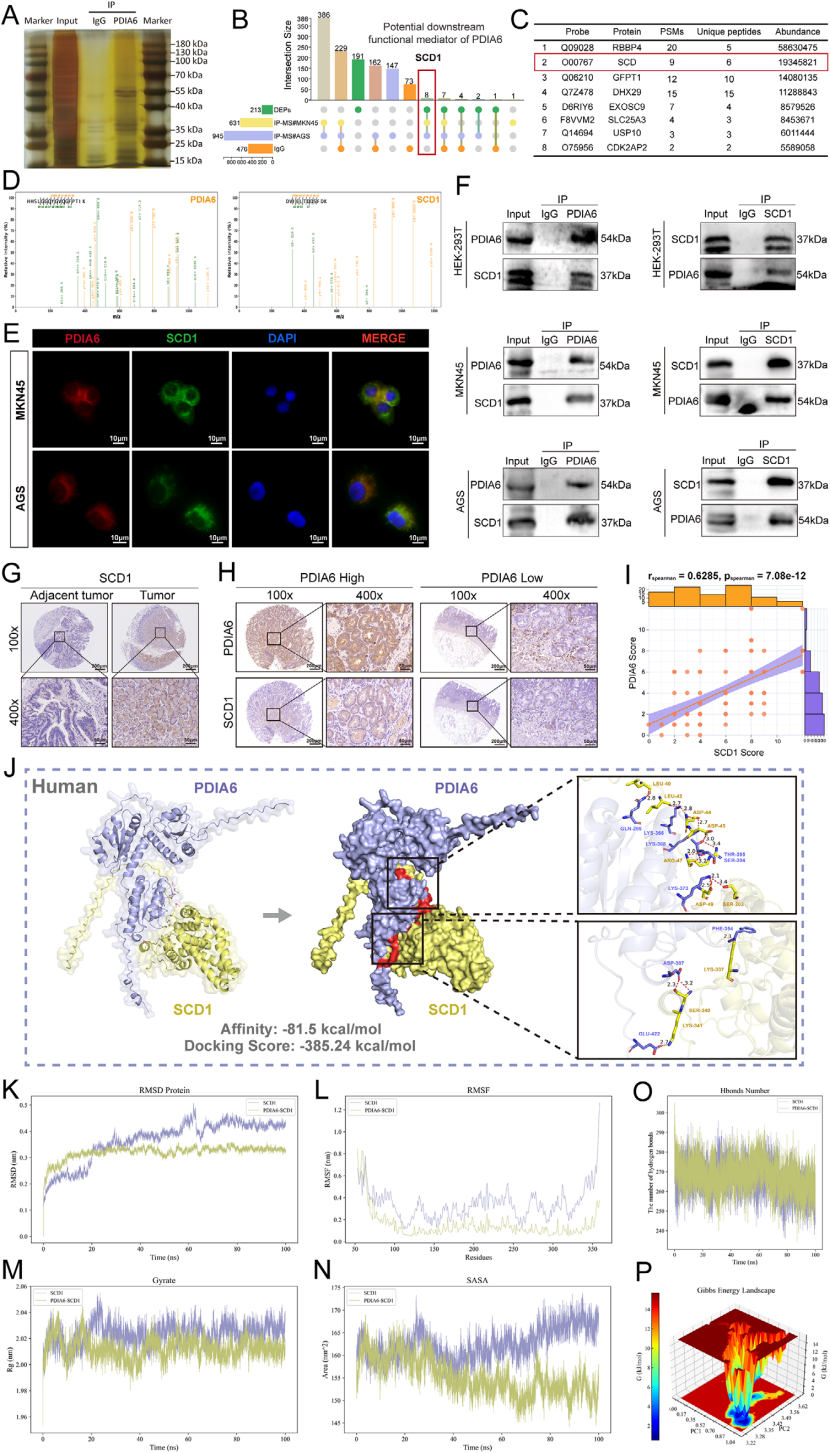
PDIA6 physically interacts with SCD1 in GC cells. (A) Silver staining of proteins co‐immunoprecipitated (CO‐IP) with PDIA6 from GC cell lysates, followed by mass spectrometry (MS) analysis. (B) UpSet plot of the overlap between PDIA6‐interacting proteins identified by IP‐MS and differentially expressed proteins from DIA‐based proteomic analysis. (C) Ranked abundance plot of candidate PDIA6‐interacting proteins. (D) Representative MS/MS spectra of PDIA6‐ and SCD1‐derived peptides identified in PDIA6 immunoprecipitates by MS. (E) Immunofluorescence staining showing cytoplasmic colocalization of PDIA6 and SCD1 in GC cells. F) Co‐IP analysis of the interaction between PDIA6 and SCD1 in HEK‐293T, MKN45, and AGS cells. (G) IHC staining of SCD1 in GC tissues and matched adjacent non‐tumor tissues. (H,I) Correlation analysis of PDIA6 and SCD1 expression in GC specimens from the same cohort. (J) Protein‐protein docking model of the predicted PDIA6‐SCD1 interaction interface. (K–P) Molecular dynamics simulation analyses of the structural stability and interaction features of the PDIA6‐SCD1 complex.

To identify the downstream effectors linking PDIA6 to lipid metabolism, co‐immunoprecipitation (Co‐IP), followed by mass spectrometry (MS) was performed to profile PDIA6‐interacting proteins (Figure 4A). Integration of PDIA6‐interacting candidates from Co‐IP/MS with DEPs identified by DIA proteomics highlighted SCD1 as the only overlapping protein directly related to lipid biosynthesis (Figure 4B,C). Representative MS/MS spectra further confirmed the presence of SCD1‐derived peptides in the PDIA6 immunoprecipitates (Figure 4D), supporting SCD1 as a candidate downstream effector. Immunofluorescence staining showed prominent cytoplasmic colocalization of PDIA6 and SCD1 in GC cells (Figure 4E), and Co‐IP assays further validated their physical association in HEK‐293T, MKN45, and AGS cells (Figure 4F). IHC analysis of GC specimens from the same cohort showed that SCD1 expression was elevated in tumor tissues relative to adjacent non‐tumor tissues and was positively correlated with PDIA6 expression (Spearman *r* = 0.6285, p < 0.0001; Figure 4G–I), thus supporting coordinated expression.

To gain structural insights into the PDIA6–SCD1 interaction, molecular docking analyses were performed. The docking model revealed a well‐defined interface between PDIA6 and SCD1, involving predicted hydrogen bonds (2.08–2.75 Å) between PDIA6 residues Lys368, Lys373, Phe354, and Glu422 and SCD1 residues Asp44, Asp49, Lys337, and Lys341 (Figure 4J; Figure S4A,B). Surface hydrophobicity mapping further identified complementary hydrophobic patches at the interface, which suggests that both polar and hydrophobic contacts contributed to complex formation (Figure 4J). Structural domain analysis also supported spatial compatibility between the two proteins, with PDIA6 containing two thioredoxin‐like catalytic domains (a and a′) and a C‐terminal non‐catalytic domain, whereas SCD1 adopts a membrane‐associated desaturase architecture with cytosol‐facing regions accessible for binding (Figure S4C,D).

Thereafter, the dynamic stability of the PDIA6‐SCD1 complex was assessed using molecular dynamics simulations (Figure 4K–P). Compared with SCD1 alone, the PDIA6–SCD1 complex exhibited reduced conformational fluctuations, as reflected by lower root mean square deviation (RMSD, 0.32 ± 0.03 nm vs. 0.36 ± 0.08 nm) and root mean square fluctuation (RMSF, 0.15 ± 0.13 nm vs. 0.34 ± 0.15 nm) values (Figure 4K,L; Figure S4E). The complex maintained a stable radius of gyration (2.01 ± 0.01 nm vs. 2.04 ± 0.01 nm) and reduced solvent‐accessible surface area (155.21 ± 4.09 nm^2^ vs. 162.48 ± 3.51 nm^2^), suggesting increased structural compactness upon PDIA6 binding (Figures 4M,N; Figure S4E). Furthermore, the complex sustained increased intermolecular hydrogen bonds (275 ± 8 vs. 266 ± 8) and converged to a single dominant minimum in the Gibbs free energy landscape, indicating an energetically favorable and dynamically stable complex (Figures 4O,P; Figure S4E). Collectively, these data support a model wherein PDIA6 physically associates with SCD1 to form a stable complex, thereby providing a structural basis for the regulation of SCD1 stability and function.

### 2.5 PDIA6 Regulates SCD1 Stability and Ubiquitination

**FIGURE 5:**
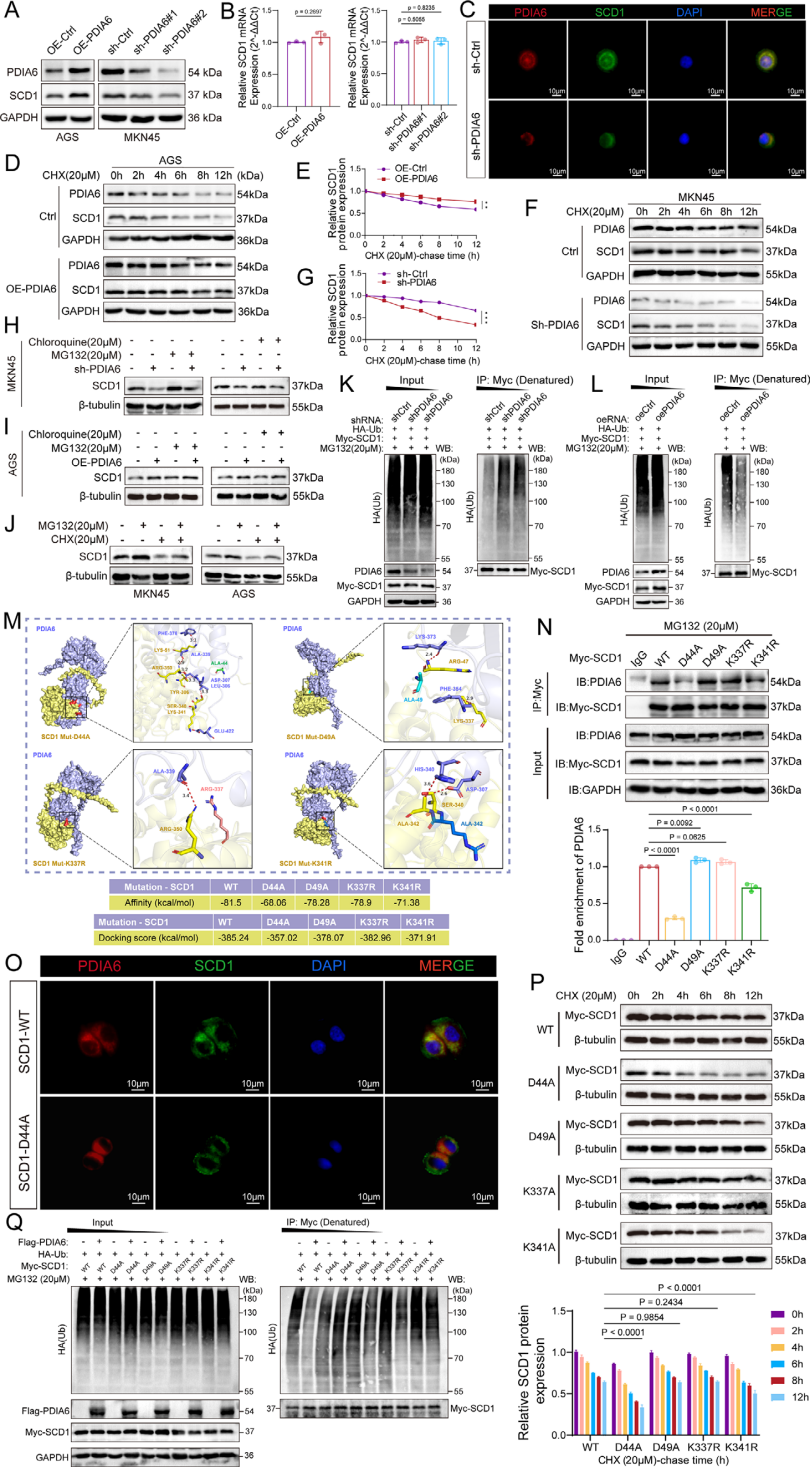
PDIA6 regulates SCD1 stability and ubiquitination. (A,B) Western blot and qRT‐PCR analyses of SCD1 protein and mRNA levels in PDIA6‐knockdown MKN45 cells and PDIA6‐overexpressing AGS cells. (C) Immunofluorescence staining of SCD1 in PDIA6‐knockdown MKN45 cells. (D–G) CHX (20 µM) chase assays in PDIA6‐knockdown MKN45 cells and PDIA6‐overexpressing AGS cells. (H,I) Western blot analysis of SCD1 protein levels in PDIA6‐knockdown MKN45 cells and PDIA6‐overexpressing AGS cells after treatment with MG132 (20 µM, 6 h) or chloroquine (20 µM, 12 h). (J) Western blot analysis of SCD1 protein levels in GC cells treated with CHX (20 µM, 12 h), with or without MG132 (20 µM) added during the final 6 h. (K,L) Immunoprecipitation analysis of SCD1 polyubiquitination in HEK‐293T cells co‐transfected with Myc‐SCD1, HA‐ubiquitin, and the indicated PDIA6 overexpression or knockdown constructs. Cells were pretreated with MG132 (20 µM, 6 h) before harvest. (M) Structure‐guided virtual residue scanning identifying candidate SCD1 residues involved in PDIA6 binding. (N) Co‐IP analysis of PDIA6 interaction with SCD1 point mutants (D44A, D49A, K337R, K341R) in MKN45 cells. (O) Immunofluorescence analysis of PDIA6 colocalization with SCD1 mutants in MKN45 cells. (P) CHX (20 µM) chase assay of SCD1 mutant stability in MKN45 cells. (Q) Polyubiquitination analysis of wild‐type and mutant SCD1 in HEK‐293T cells co‐expressing Myc‐SCD1, Flag‐PDIA6, and HA‐ubiquitin after MG132 pretreatment (20 µM, 6 h). Data are presented as mean ± SD. *, *p* < 0.05; **, *p* < 0.01; ***, *p* < 0.001; and ns, not significant.

The study further investigated whether PDIA6 regulates SCD1 expression. Western blot analysis showed that PDIA6 overexpression increased SCD1 protein levels, whereas PDIA6 knockdown decreased these levels (Figure 5A). In contrast, qRT‐PCR revealed no appreciable changes in SCD1 mRNA expression (Figure 5B), indicating that PDIA6 primarily regulates SCD1 expression at the post‐translational level. Consistently, immunofluorescence staining confirmed reduced cytoplasmic SCD1 signal upon PDIA6 knockdown (Figure 5C).

Cycloheximide (CHX) chase assays were performed to determine whether PDIA6 affects SCD1 protein stability. Quantification of western blot signals showed that SCD1 declined progressively during CHX (20 µM) treatment in both cell models, with degradation kinetics strongly influenced by PDIA6 expression. In AGS cells, PDIA6 overexpression delayed SCD1 degradation, whereas in MKN45 cells, PDIA6 knockdown accelerated it, thereby supporting the stabilizing effect of PDIA6 on SCD1 (Figures 5D–G). To define the pathway responsible for SCD1 degradation, PDIA6‐knockdown MKN45 cells and PDIA6‐overexpressing AGS cells were treated with the proteasome inhibitor MG132 (20 µM, 6 h) or the autophagy‐lysosome inhibitor chloroquine (CQ, 20 µM, 12 h). MG132, but not CQ, markedly restored SCD1 protein levels following PDIA6 knockdown, indicating that PDIA6 stabilizes SCD1 by protecting it from proteasome‐dependent degradation (Figures 5H,I). Furthermore, in endpoint assays shown in Figure 5J, SCD1 reduction induced by CHX (20 µM, 12 h) was partially rescued by MG132 (20 µM, 6 h) co‐treatment, which further supported proteasome‐dependent degradation. Consistent with this finding, ubiquitination assays showed that PDIA6 knockdown increased Myc‐SCD1 polyubiquitination, whereas PDIA6 overexpression decreased it (Figures 5K,L). Notably, the comparable Myc‐SCD1 input levels across groups under MG132 treatment (20 µM, 6 h) are likely attributable to the blockade of proteasome‐dependent degradation, which leads to the accumulation of polyubiquitinated SCD1. Moreover, overexpression of exogenous Myc‐SCD1 may have partially masked differences in steady‐state protein abundance.

To define the structural basis of this regulation, structure‐guided virtual residue scanning and molecular docking analyses were performed. These analyses identified Asp44, Asp49, Lys337, and Lys341 of SCD1 as candidate interface residues involved in PDIA6 binding (Figure 5M). Binding free energy calculations revealed that alanine substitution at Asp44 (D44A) resulted in a pronounced loss of predicted binding affinity toward PDIA6 (ΔΔG ≈ +13 kcal/mol relative to wild type), whereas substitution at Lys341 had an intermediate effect and substitutions at Asp49 or Lys337 had weaker effects. Docking score analyses showed a concordant trend, with D44A mutation displaying the most pronounced reduction in docking stability. Consistent with these predictions, Co‐IP assays demonstrated that the D44A mutation markedly impaired the PDIA6–SCD1 interaction, whereas K341R moderately weakened binding, and D49A or K337R had minimal effects (Figure 5N; Table S3). Immunofluorescence analysis further confirmed the reduced colocalization of PDIA6 and the SCD1‐D44A mutant (Figure 5O). Functionally, CHX chase assays revealed that the D44A mutation markedly accelerated SCD1 degradation (Figure 5P), and ubiquitination assays further demonstrated enhanced polyubiquitination of the mutant relative to that of wild‐type SCD1 (Figure 5Q). Collectively, these results indicate that PDIA6 stabilizes SCD1 by restraining its ubiquitin‐proteasome‐mediated degradation, with Asp44 serving as a critical structural determinant linking PDIA6 binding to SCD1.

### 2.6 SCD1 Functionally Mediates PDIA6‐Driven Lipid Metabolic Remodeling and Malignant Phenotypes

**FIGURE 6:**
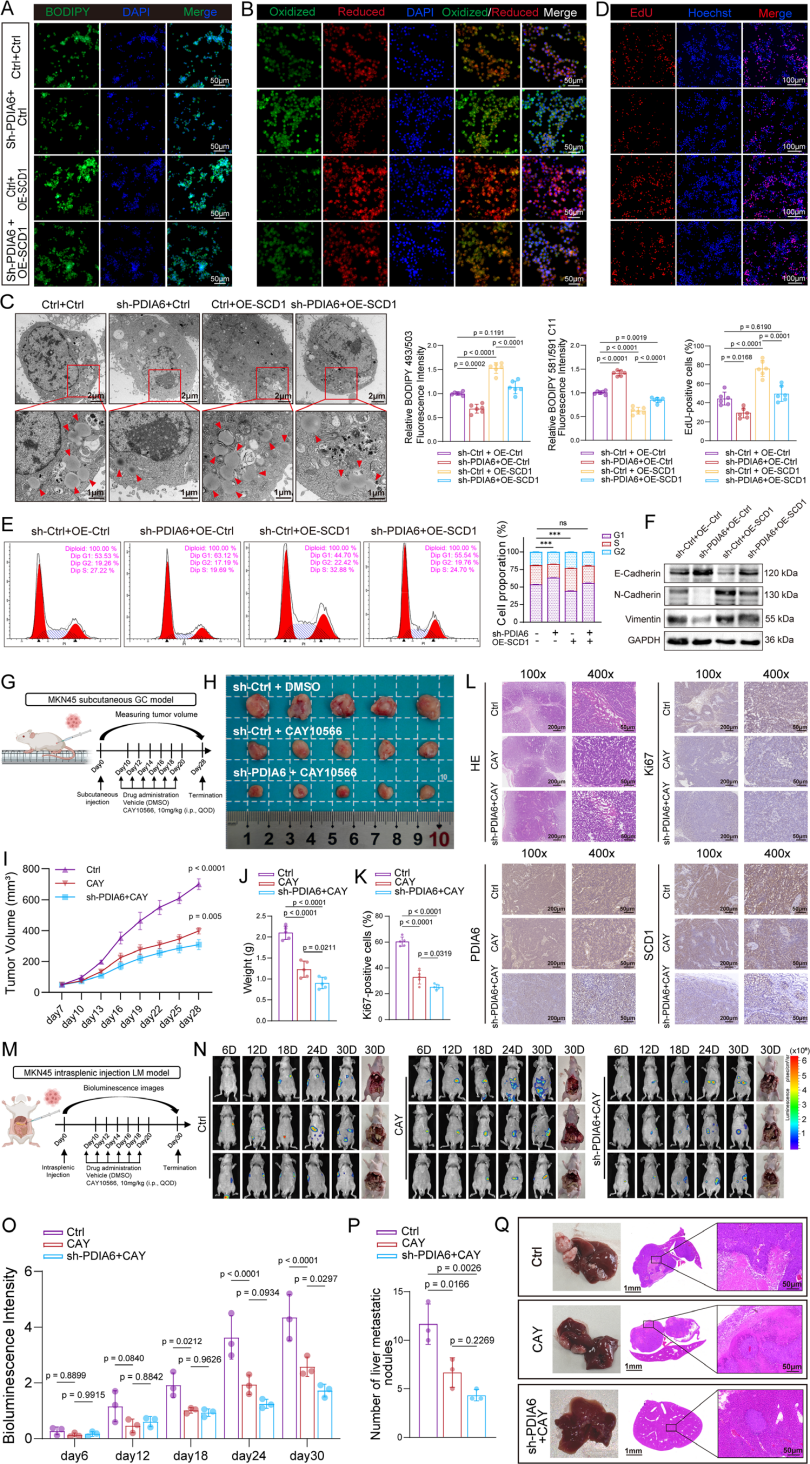
SCD1 mediates PDIA6‐driven oncogenic phenotypes in GC. (A) R Representative images and quantification of neutral lipid accumulation assessed by BODIPY 493/503 staining. (B) Representative images and quantification of lipid peroxidation measured by BODIPY 581/591 C11 staining. (C) Transmission electron microscopy of lipid droplets (red arrows). (D) Representative images and quantification of EdU incorporation assays. (E) Flow cytometric analysis of cell‐cycle distribution and quantification of the percentages of cells in G1, S, and G2 phases. (F) Western blot analysis of EMT‐related markers. (G) Schematic illustration of the subcutaneous xenograft model and treatment regimen. (H,J) Representative images, tumor growth curves, and tumor weights of xenografts derived from control or PDIA6‐knockdown MKN45 cells treated with or without CAY10566 (10 mg/kg, i.p., QOD) (n = 5 mice per group). (K) Quantification of Ki67‐positive cells in xenograft tumors from the indicated treatment groups. L) Representative H&E images and IHC staining for Ki67, PDIA6, and SCD1 in xenograft tissues (100× and 400×). (M) Schematic illustration of the splenic injection liver metastasis model and treatment regimen. (N–Q) Fluorescence imaging, gross liver images, quantification of metastatic burden, and H&E staining of liver metastatic lesions derived from control or PDIA6‐knockdown MKN45 cells treated with or without CAY10566 (10 mg/kg, i.p., QOD) (n = 3 mice per group). For in vitro assays shown in A–F, experiments were performed in PDIA6‐knockdown MKN45 cells with or without SCD1 re‐expression. Data are presented as mean ± SD. *, *p* < 0.05; **, *p* < 0.01; ***, *p* < 0.001; and ns, not significant. [Correction added on 17 June 2026, after first online publication: Figure 6L has been updated in this version.]

To determine whether SCD1 functionally mediates PDIA6‐driven lipid metabolic remodeling and malignant phenotypes, rescue experiments were performed in PDIA6‐knockdown MKN45 cells. Re‐expression of SCD1 partially restored neutral lipid accumulation and attenuated lipid peroxidation induced by PDIA6 knockdown, as shown by BODIPY 493/503 staining, BODIPY 581/591 C11 staining, and transmission electron microscopy (Figures 6A–C). Functionally, SCD1 re‐expression partially rescued the defects in DNA synthesis, S‐phase entry, cell migration, and EMT marker expression induced by PDIA6 knockdown (Figures 6D–F; Figure S5A,B). These results indicate that SCD1 is a key downstream effector of PDIA6 in the regulation of both lipid metabolism and malignant phenotypes.

To further establish the causal relationship within the PDIA6–SCD1 axis, we performed complementary pharmacological inhibition and metabolic rescue experiments. In PDIA6‐overexpressing AGS cells, treatment with the selective SCD1 inhibitor CAY10566 (1 µM, 24 h) markedly attenuated PDIA6‐induced cell proliferation, migration, and intracellular neutral lipid accumulation, while concomitantly increasing lipid peroxidation (Figure S6A–E). These findings demonstrate that the oncogenic effects of PDIA6 are mediated, at least in part, through SCD1 enzymatic activity. We next conducted targeted metabolic rescue experiments to test whether the downstream lipid metabolic output is functionally required for these phenotypes. Exogenous oleic acid (OA) supplementation (10 µM, 24 h) partially reversed the inhibitory effects of CAY10566 (1 µM, 24 h) on neutral lipid accumulation and malignant phenotypes in PDIA6‐overexpressing AGS cells (Figure S6A–E). Reciprocally, in PDIA6‐knockdown MKN45 cells, exogenous OA supplementation (10 µM, 24 h) partially rescued the impaired proliferative and migratory capacities, restored neutral lipid levels, and attenuated elevated lipid peroxidation (Figure S7A–E). Collectively, these bidirectional functional experiments provide robust evidence that lipid metabolic reprogramming downstream of the PDIA6–SCD1 axis drives GC cell adaptation and malignant progression.

To further evaluate the in vivo relevance of this axis, the effects of CAY10566 (10 mg/kg, intraperitoneal [i.p.], QOD) on a PDIA6‐deficient mouse model were tested. In subcutaneous xenograft models (n = 5 mice per group), CAY10566 monotherapy substantially suppressed tumor growth, whereas the combination of PDIA6 knockdown and CAY10566 treatment produced stronger inhibition than that under either treatment alone, with greater reductions in tumor volume, tumor weight, and Ki67 positivity (Figure 6G–L). Similarly, in the splenic injection liver metastasis model (n = 3 mice per group), CAY10566 attenuated hepatic metastatic burden, whereas the combination treatment produced the most pronounced suppression, as evidenced by the fewest and smallest hepatic metastatic lesions (Figure 6M–Q). No overt systemic toxicity was observed during the treatment. Collectively, these findings suggest that PDIA6 promotes GC progression, at least in part, through SCD1‐dependent lipid metabolic regulation and that PDIA6 deficiency enhances sensitivity to SCD1 inhibition.

### 2.7 STAT3 Regulates PDIA6 Transcription in GC

**FIGURE 7:**
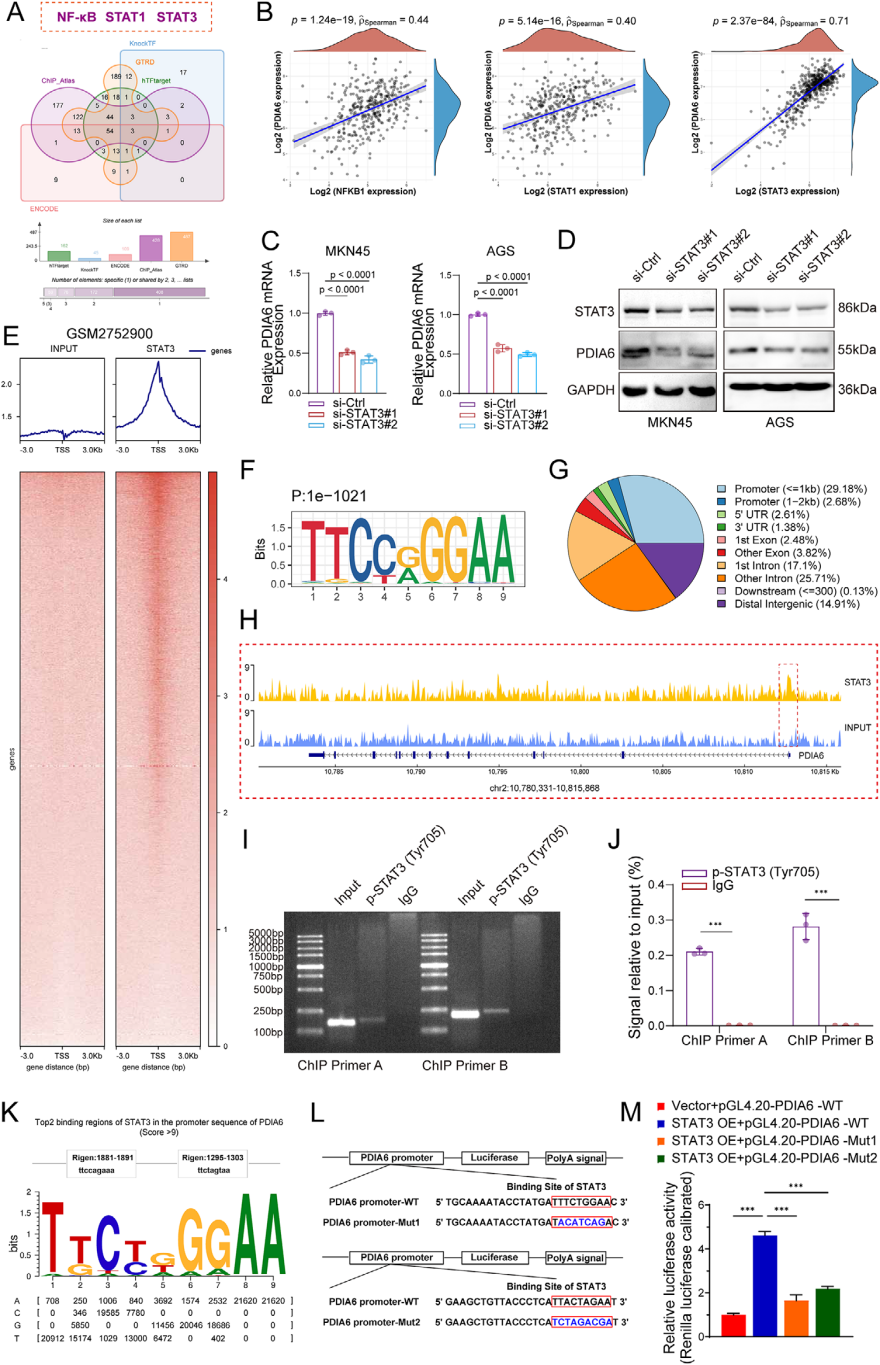
STAT3 transcriptionally upregulates PDIA6 in GC. A) Integrative database analyses identifying NF‐κB, STAT1, and STAT3 as candidate transcription factors regulating PDIA6. B) Correlation analysis between PDIA6 expression and candidate transcription factors in the TCGA‐STAD cohort. C,D) qRT‐PCR and western blot analyses of PDIA6 expression after siRNA‐mediated STAT3/STAT1 knockdown or NF‐κB inhibition (BAY117082) in MKN45 and AGS cells. E) Heatmap of STAT3 ChIP‐seq signal distribution around transcription start sites. F) Motif enrichment analysis of STAT3 ChIP‐seq peaks. G) Genomic annotation of STAT3 ChIP‐seq peaks by ChIPSeeker. H) STAT3 ChIP‐seq peak profile across the PDIA6 promoter region. I) Schematic illustration of predicted STAT3‐binding sites within the PDIA6 promoter based on JASPAR analysis. J,K) ChIP‐PCR and ChIP‐qPCR assays using anti‐phospho‐STAT3 (Tyr705) antibody or IgG control to assess STAT3 occupancy at the PDIA6 promoter. L,M) Dual‐luciferase reporter assays evaluating the activity of wild‐type or STAT3‐binding‐site‐mutant PDIA6 promoters after STAT3 overexpression. Data are presented as mean ± SD. *, *p* < 0.05; **, *p* < 0.01; ***, *p* < 0.001; and ns, not significant.

Given the pronounced upregulation of PDIA6 in GC, the study investigated whether aberrant transcriptional regulation contributes to its expression. Integrated in silico analyses using ChIP_Atlas, KnockTF, hTFtarget, ENCODE, and GTRD nominated nuclear factor‐kappa B (NF‐κB), STAT1, and STAT3 as candidate transcription factors with predicted occupancy at the PDIA6 locus (Figure 7A). TCGA‐STAD correlation analysis showed strong positive association between PDIA6 and STAT3, with weak correlations for NF‐κB and STAT1 (Figure 7B). Consistently, small interfering‐RNA‐mediated STAT3 knockdown markedly reduced PDIA6 expression at both the mRNA and protein levels, whereas pharmacological inhibition of NF‐κB using BAY117082 or STAT1 knockdown had minimal effects (Figures 7C,D; Figure S5C,D), thereby indicating a dominant role for STAT3 in PDIA6 regulation.

Subsequently, the direct effects of STAT3 on PDIA6 transcription were tested. Analysis of publicly available STAT3 ChIP‐seq data from AGS cells (GSM2752900) showed pronounced STAT3 enrichment around the transcription start sites (TSSs), which was absent in the input controls (Figure 7E). Motif enrichment analysis identified a highly critical consensus sequence matching the canonical STAT3 binding motif (TTCCGGGAA; p = 1 × 10^−^^10^ ^2^ ^1^) (Figure 7F). ChIPSeeker annotation further showed that approximately 30% of STAT3 binding events were localized within promoter regions (≤1 kb) (Figure 7G), including a prominent peak at the PDIA6 promoter (Figure 7H). Direct promoter occupancy was further confirmed via ChIP‐PCR and ChIP‐qPCR, which showed pronounced enrichment of PDIA6 promoter fragments in phospho‐STAT3 (Tyr705) immunoprecipitates but not in IgG controls (Figure 7I,J). JASPAR predicted two putative STAT3 binding motifs within the PDIA6 promoter region (Figure 7K). Dual‐luciferase reporter assays demonstrated that STAT3 overexpression markedly enhanced wild‐type PDIA6 promoter activity, whereas mutation of the predicted STAT3‐binding sites abolished this effect (Figure 7L,M). Collectively, these results indicated that activated STAT3 directly binds to the PDIA6 promoter and transcriptionally upregulates PDIA6 in GC cells.

### 2.8 Contribution of Tumor Microenvironmental Cues to PDIA6 Upregulation

**FIGURE 8:**
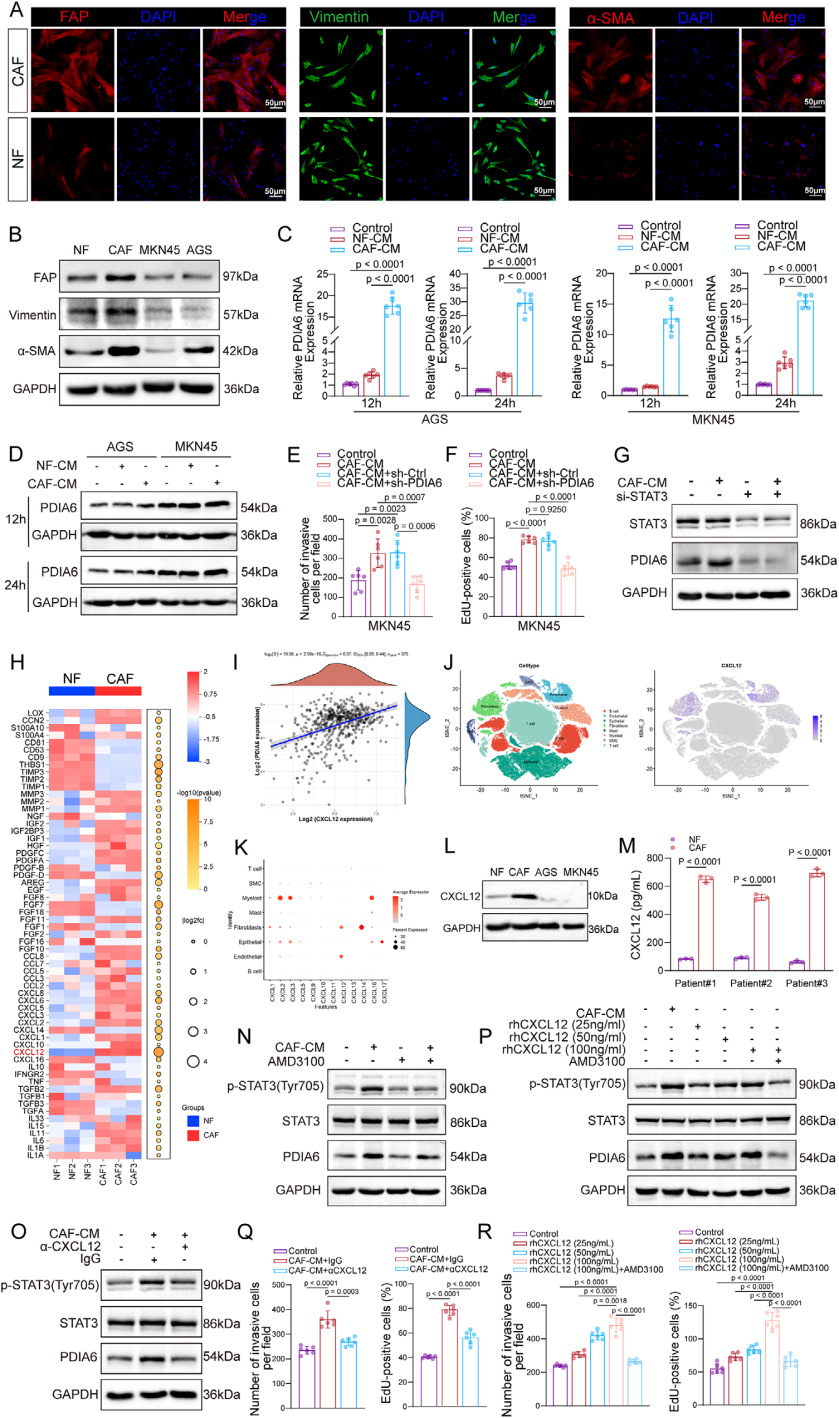
CAF‐derived CXCL12 reinforces PDIA6 expression through CXCR4‐STAT3 signaling in GC. (A,B) Immunofluorescence staining and western blot analysis of the CAF markers FAP and α‐SMA in CAFs and paired NFs. (C,D) qRT‐PCR and western blot analyses of PDIA6 mRNA and protein expression in GC cells treated with CAF‐conditioned medium (CAF‐CM) or NF‐conditioned medium (NF‐CM). (E,F) Transwell migration and EdU incorporation assays evaluating the effects of PDIA6 knockdown on CAF‐CM‐induced GC cell phenotypes. (G) Western blot analysis of STAT3 and PDIA6 in GC cells treated with or without CAF‐CM and transfected with control or STAT3 siRNA. (H) RNA‐seq analysis identifying CXCL12 as a highly upregulated secreted factor in CAFs relative to NFs. (I) Correlation analysis of CXCL12 and PDIA6 expression in the TCGA‐STAD cohort. (J,K) Single‐cell RNA‐seq analysis showing fibroblast‐specific expression of CXCL12 in GC tissues. (L,M) Western blot analysis of CXCL12 protein expression and ELISA of CXCL12 secretion in CAFs, NFs, and GC cells. (N) Western blot analysis of p‐STAT3 (Tyr705), total STAT3, and PDIA6 in GC cells pretreated with AMD3100 (2.5 µg/mL) for 30 min and then incubated with or without CAF‐CM for 24 h. (O) Western blot analysis of p‐STAT3 (Tyr705), total STAT3, and PDIA6 in GC cells treated with CAF‐CM together with IgG control or αCXCL12 (10 µg/mL, 24 h). (P) Western blot analysis of p‐STAT3 (Tyr705) and PDIA6 in GC cells treated with CAF‐CM or recombinant human CXCL12 (rhCXCL12, 25/50/100 ng/mL) for 24 h, with or without AMD3100 pretreatment. (Q,R) Cell proliferation and migration assays evaluating the effects of rhCXCL12 stimulation and αCXCL12 neutralization on GC cell behavior. Data are presented as mean ± SD.

Given the identification of STAT3 as a direct transcriptional regulator of PDIA6, the study examined whether tumor microenvironmental cues contributed to PDIA6 upregulation. Cancer‐associated fibroblasts (CAFs) and paired normal fibroblasts (NFs) were isolated from fresh GC specimens and phenotypically validated (Figure S8A). CAFs showed markedly higher expression of fibroblast activation protein (FAP) and α‐smooth muscle actin (α‐SMA) than that in NFs (Figure 8A,B). Moreover, these activated features were maintained during in vitro expansion. CAF‐conditioned medium (CM) increased PDIA6 expression in GC cells at both the mRNA and protein levels, whereas NF‐CM did not (Figure 8C,D). Functionally, PDIA6 knockdown attenuated the proliferative and migratory effects of CAF‐CM (Figure 8E,F). Importantly, the CAF‐CM induced upregulation of PDIA6 was abolished upon STAT3 silencing, thereby supporting the requirement for STAT3 activation in this response (Figure 8G).

To identify the CAF‐derived factors responsible for PDIA6 induction, the secreted genes between matched CAFs and NFs were compared, and these data were integrated with TCGA‐STAD transcriptomic analysis. This approach highlighted CXCL12 as a CAF‐enriched secreted factor strongly associated with PDIA6 expression (Figure 8H,I; Figure S8B). Analysis of an independent GC scRNA‐seq dataset (GSE183904) further showed that CXCL12 is predominantly expressed in fibroblast populations within tumor tissues (Figure 8J,K; Figure S8C). Consistent with this, western blot analysis showed high CXCL12 protein expression in CAFs (Figure 8L), whereas enzyme‐linked immunosorbent assay (ELISA) confirmed abundant CXCL12 secretion, with minimal levels detected in NFs and GC cells (Figure 8M).

Considering the established role of CXCL12–CXCR4 signaling in STAT3 activation, its involvement in CAF‐mediated PDIA6 regulation was examined. Pretreatment with the CXCR4 inhibitor AMD3100 (2.5 mg/L, 30 min), followed by 24 h incubation with CAF‐CM, suppressed STAT3 phosphorylation at Tyr705 and concomitant PDIA6 upregulation (Figure 8N). Similarly, CXCL12 neutralization with αCXCL12 (10 µg/mL), maintained during 24 h incubation with CAF‐CM, attenuated phosphorylated‐STAT3 induction and PDIA6 expression (Figure 8O). Conversely, recombinant human CXCL12 (rhCXCL12, 100 ng/mL, 24 h) partially recapitulated the effects of CAF‐CM, inducing STAT3 phosphorylation and PDIA6 expression (Figure 8P). Total STAT3 levels remained unchanged under these conditions, which indicated that PDIA6 induction was driven by STAT3 activation rather than by altered STAT3 abundance. Functionally, rhCXCL12 enhanced GC cell proliferation and migration, whereas αCXCL12 impaired CAF‐CM‐mediated effects (Figures 8Q,R). Consistent with these findings, TCGA‐STAD analysis revealed positive correlations between PDIA6 expression and the components of the CXCL12/CXCR4–STAT3 axis (Figure 7B; Figure S8D), thus supporting the clinical relevance of this stromal‐tumor signaling pathway.

## 3 Discussion

**FIGURE 9:**
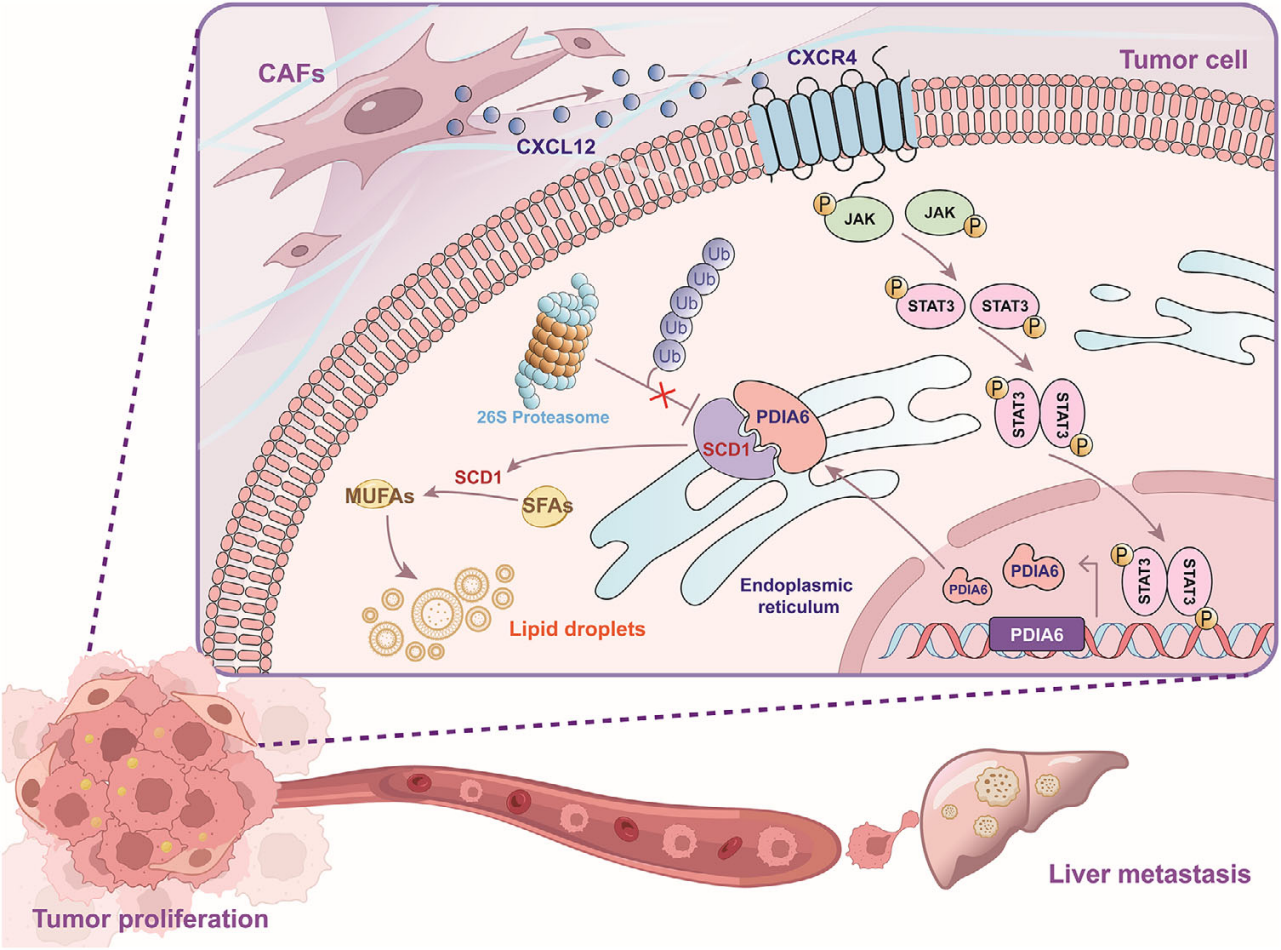
Schematic model illustrating the PDIA6–SCD1 axis linking tumor–stromal signaling to lipid metabolic reprogramming and GC progression.

PDIA6 has recently gained attention in cancer biology [6, 9, 31, 32, 33], yet its role in GC remains poorly understood. In this study, PDIA6 was identified as a key regulator of lipid metabolic reprogramming that promotes GC growth and metastasis (Figure 9). Integrative analyses across TCGA, Gene Expression Omnibus (GEO), and multi‐omics datasets showed that PDIA6 is upregulated in GC and is associated with advanced disease and poor prognosis. Functionally, PDIA6 knockdown induced coordinated lipid remodeling, which was characterized by reduced lipid droplet accumulation, enhanced lipid peroxidation, and suppression of malignant phenotypes. Mechanistically, PDIA6 interacted with SCD1 to sustain lipid desaturation and preserve MUFA‐enriched neutral lipid homeostasis, thereby maintaining the metabolic fitness of GC cells. Collectively, these findings expand the functional landscape of PDIA6 beyond ER proteostasis and redox control. Specifically, this study establishes PDIA6 as a previously unrecognized metabolic requirement in GC and reveals the PDIA6–SCD1 axis as a therapeutically relevant vulnerability.

The central mechanistic finding of this study was that PDIA6 stabilized SCD1 at the protein level. As the rate‐limiting enzyme for MUFA biosynthesis, SCD1 is a key determinant of membrane plasticity and oxidative stress tolerance in cancer cells [20, 34, 35, 36]. Previous studies have mainly focused on the transcriptional regulation of SCD1. This includes ADAR1‐mediated A‐to‐I editing, which enhances KHDRBS1 binding [35], as well as circTFRC‐mediated recruitment of ELAVL1 [36] to stabilize SCD1 transcripts. However, the current study uncovered a distinct post‐translational mechanism. Structural docking, molecular dynamics simulations, mutational analysis, and biochemical assays converged on Asp44 within the N‐terminal cytosolic region of SCD1 as a critical determinant of PDIA6 binding. Disruption of this interface weakened the PDIA6‐SCD1 interaction and reduced SCD1 stability, thereby supporting a direct role of PDIA6 binding in maintaining SCD1 abundance. This interaction is mechanistically plausible, considering the chaperone‐like properties of PDIA6 and the accessible cytosolic termini of the ER‐resident multipass enzyme SCD1 [7, 32, 37, 38, 39, 40, 41]. These findings support a structure‐based mechanism by which PDIA6 protects SCD1 from degradation and maintains lipid metabolic homeostasis.

Notably, the basal degradation of PDIA6 and SCD1 was slower in control MKN45 cells than in control AGS cells during CHX treatment, suggesting cell‐line‐specific differences in intrinsic protein turnover. Given that our study establishes PDIA6 as a direct stabilizer of SCD1, these observed inter‐cell‐line variations in PDIA6 stability likely propagate to affect SCD1 degradation kinetics. Specifically, the higher steady‐state abundance and prolonged half‐life of PDIA6 in MKN45 cells relative to AGS cells may account for the attenuated basal degradation of SCD1 observed in this cell line. Considering the molecular heterogeneity of GC and the context‐dependence of proteostasis networks, this interpretation is biologically plausible [42, 43, 44]. Potential mechanisms underlying the enhanced stability of PDIA6 in MKN45 cells include differential ER‐associated degradation (ERAD) activity, distinct post‐translational modifications of PDIA6, or variations in the expression of PDIA6‐interacting cofactors [45, 46, 47], however, these mechanistic hypotheses were not directly addressed in the current study. Critically, these cell‐line‐specific differences do not compromise the central conclusion of our work, as PDIA6 modulation consistently and robustly altered SCD1 degradation kinetics within each individual cell line.

Although our data indicate that PDIA6 stabilizes SCD1 by suppressing its polyubiquitination and proteasomal degradation, the responsible E3 ubiquitin ligase remains undefined. As a short‐lived ER‐resident protein, SCD1 undergoes turnover through its N‐terminal PEST degron [48, 49] and may also be subject to ERAD, in which HRD1 and gp78 represent plausible ERAD‐associated candidates [50, 51]. Notably, BioGRID and STRING predict an interaction between PDIA6 and SEL1L, a core adaptor of the HRD1 ERAD complex, suggesting a potential link between PDIA6 and ERAD‐associated control of SCD1 stability. Our data are most consistent with a model in which direct PDIA6–SCD1 binding restrains SCD1 ubiquitination. In particular, PDIA6 bound SCD1 at Asp44, a residue located near the N‐terminal PEST degron, and mutation of Asp44 disrupted this interaction while increasing SCD1 polyubiquitination and degradation. These findings support a protection‐by‐binding mechanism, whereby PDIA6 may sterically shield the degron or alter its local conformation to limit access of ERAD‐associated ubiquitination machinery. Notably, PDIA6 has also been reported to protect TRAF4 from SMURF1‐mediated ubiquitination in esophageal cancer [52], supporting the plausibility of such a mechanism. This model is conceptually analogous to established protection‐by‐binding paradigms such as p21‐PCNA [53, 54] and Nrf2‐p62 [55]. In addition, the documented chaperone activity of PDIA6 [56], together with our molecular dynamics simulations showing increased SCD1 compactness upon PDIA6 binding, raises the possibility that PDIA6 may also reduce SCD1 susceptibility to ER quality‐control surveillance by promoting conformational stability. Collectively, these observations support a model in which PDIA6 stabilizes SCD1 through direct interaction, while the precise contribution of ERAD components, including the relevant E3 ligase(s), remains to be defined.

Beyond tumor cell‐intrinsic signaling, our in vitro data suggest that PDIA6 expression may also be modulated by cues from the TME. While pan‐cancer analyses showed widespread PDIA6 upregulation across malignancies (Figure S8E), its upstream regulators remain unclear. In GC, we identified STAT3 as a direct transcriptional regulator of PDIA6, which can be activated by various stromal signals [23, 25, 57, 58, 59, 60]. CAFs are the dominant stromal population in GC [26, 61, 62], and our secretome analysis linked CAF‐derived CXCL12 to PDIA6 expression. Specifically, CXCL12 engagement with its receptor CXCR4 triggers STAT3 phosphorylation and nuclear translocation, leading to robust PDIA6 transcriptional induction. Given that STAT3 integrates multiple extracellular signals, the CXCL12/CXCR4 pathway is likely one of several upstream regulators of PDIA6. Therefore, these findings support a contributory rather than definitive role for the CAF–CXCL12–STAT3 axis in PDIA6 activation.

From a translational perspective, this study uncovers PDIA6–SCD1‐dependent metabolic vulnerability in GC. Although SCD1 inhibition alone has shown antitumor activity in preclinical models, its effects are often context‐dependent. In the current study models, combined PDIA6 knockdown and SCD1 inhibition produced a stronger suppression of tumor growth and metastasis than that under either monotherapy, thereby indicating a heightened dependence of PDIA6‐deficient tumors on SCD1‐driven lipid desaturation. This raises the possibility of PDIA6 expression serving as a prognostic and predictive biomarker to stratify patients likely to respond to SCD1‐targeted or combination metabolic therapy.

Several limitations should be acknowledged. First, the molecular mechanism by which PDIA6 limits SCD1 ubiquitination and degradation remains incompletely elucidated. Although our data support a direct interaction‐based model, the specific E3 ubiquitin ligase and alternative regulatory mechanisms remain to be clarified. Subsequent research aims to resolve this gap and elaborate this regulatory pathway. Second, the proposed CAF–CXCL12–STAT3 upstream axis remains to be validated in vivo, and the mouse models employed in this study cannot fully recapitulate the TME‐driven metabolic remodeling in human GC. Therefore, the principal mechanistic conclusion of this study is centered on the PDIA6–SCD1 axis, and more physiologically relevant models, such as orthotopic tumors, genetically engineered mouse models (GEMMs), or patient‐derived organoids, will be required to establish the in vivo contribution of this upstream pathway. Furthermore, the lack of specific PDIA6 inhibitors limits direct therapeutic validation.

## 4 Conclusions

In conclusion, this study identified PDIA6 as a key regulator of lipid metabolic reprogramming in GC. PDIA6 binds to and stabilizes SCD1 by restraining its ubiquitin–proteasome‐dependent degradation, thereby sustaining MUFA‐enriched lipid homeostasis and driving GC growth and metastasis. PDIA6 expression is further shaped in part by tumor microenvironmental cues via the CAF‐derived CXCL12–STAT3 pathway. From a translational perspective, the PDIA6–SCD1 axis represents an actionable metabolic vulnerability, as co‐targeting PDIA6 and SCD1 synergistically suppresses GC progression relative to either intervention alone. Overall, these findings establish PDIA6 as a promising prognostic biomarker and candidate therapeutic target for GC.

## 5 Methods

### 5.1 Ethical Compliance and Tissue Specimen Acquisition

This study enrolled 96 patients with GC who underwent surgical resection at Northern Jiangsu People's Hospital between January 2018 and December 2018. Paired tumor and adjacent non‑tumor tissues were collected. Histopathological diagnoses were independently confirmed by two board‑certified pathologists, and tumor staging was performed according to the eighth edition of the American Joint Committee on Cancer (AJCC) staging system. The study was approved by the Institutional Ethics Committee of Northern Jiangsu People's Hospital (approval No. 2024ky192), and written informed consent was obtained from all participants.

### 5.2 Bioinformatics Analysis

scRNA‐seq datasets were obtained from the GEO database, including GSE163558 (primary GC: GSM5004180, GSM5004181, and GSM5004182; GC liver metastasis: GSM5004188 and GSM5004189; adjacent non‐tumoral tissue: GSM5004183) and GSE183904. GSE163558 was analyzed using the Seurat package (v3) in R. Tumor‐associated epithelial cells were extracted for differential expression analysis. Upregulated genes derived from scRNA‐seq were intersected with bulk RNA‐seq data from TCGA‐STAD and GSE56807 to define a consensus tumor‐associated gene set, which was then clustered using the MCL. Cluster activity was quantified by ssGSEA, and survival associations were evaluated by Cox regression in GSE84426 and validated in TCGA‐STAD. GSE183904 was independently analyzed to assess CXCL12 expression across cell types.

### 5.3 Cell Culture

HEK293T cells (Procell, Wuhan, China) were cultured in Dulbecco's Modified Eagle Medium (DMEM; Gibco, USA). Human gastric cancer cell lines (AGS, BGC823, HGC27, Hs746T, MKN1, MKN45, N3, N87, and SNU719) and the non‐malignant human gastric epithelial cell line GES‐1 (Procell, China) were maintained in RPMI‐1640 medium (Gibco, USA). All media were supplemented with 10% fetal bovine serum (FBS; Gibco, USA) and 1% penicillin–streptomycin. Cells were cultured at 37°C in a humidified atmosphere containing 5% CO_2_.

CAY10566 was dissolved in DMSO and used at a final concentration of 1 µM, with the final DMSO concentration maintained below 0.1% in all groups. Oleic acid (OA) was prepared as a complex with fatty acid‐free bovine serum albumin (BSA) and used at a final concentration of 10 µM. For SCD1 inhibition experiments in AGS cells, CAY10566 was applied for 24 h. For metabolic rescue experiments in PDIA6‐overexpressing AGS cells, cells were pretreated with CAY10566 for 4–6 h, followed by OA supplementation without washout for a further 24 h. For rescue experiments in PDIA6‐knockdown MKN45 cells, OA was added for 24 h. Vehicle controls were included accordingly, and DMSO and BSA concentrations were matched across conditions.

### 5.4 Isolation and Culture of NFs and CAFs

NFs and CAFs were isolated from freshly resected adjacent non‐tumorous tissues and primary GC specimens, respectively, obtained from patients who had not received any preoperative anti‐tumor therapy. Samples were washed with ice‐cold phosphate‐buffered saline (PBS), minced into approximately 5 mm fragments, and digested with 1 mg/mL type IV collagenase (C8160, Solarbio, China) at 37°C for 1 h. Digestion was terminated with DMEM containing 10% fetal bovine serum (FBS), and the cell suspension was passed through a 100 µm sterile cell strainer. Residual tissue fragments were then transferred to T25 culture flasks and cultured in DMEM supplemented with 20% FBS to allow fibroblast outgrowth. Primary fibroblasts were maintained for fewer than 10 passages in DMEM containing 10% FBS and 1% penicillin–streptomycin (P/S). Expression of fibroblast activation protein (FAP), α‐smooth muscle actin (α‐SMA), and vimentin in CAFs and NFs was examined by immunofluorescence staining and western blotting. Antibodies used in this study are listed in Table S4.

### 5.5 Plasmid Construction, Small Interfering RNA, and Lentivirus Transfection

Lentiviral vectors encoding short hairpin RNAs (shRNAs) targeting PDIA6 (sh‐PDIA6_1, 5′‐GGTCACTGTCAAAGATTAACA‐3′; sh‐PDIA6_2, 5′‐GCGAGTCTCCTGTGGATTATG‐3′; sh‐PDIA6_3, 5′‐GCAAGGCATCAACGAGTTTCT‐3′), together with lentiviral constructs for PDIA6 overexpression (pLVX‐CMV‐PDIA6‐3FLAG‐WPRE‐PGK‐PURO) and SCD1 overexpression (pLVX‐CMV‐SCD1‐3FLAG‐WPRE‐PGK‐PURO), were provided by Nanjing Biotech and Pharmaceutical Valley (BPV) Biotechnology Co., Ltd. (China). For rescue experiments, stable PDIA6‐knockdown MKN45 cells were further transduced with lentiviruses encoding SCD1 or the corresponding empty vector. Cells were maintained for 72 h after infection and then selected with puromycin (2 µg/mL) to establish stable cell populations. All reagents used for lentiviral packaging and infection were obtained from the same manufacturer.

Small interfering RNAs (siRNAs) targeting STAT3 and STAT1 were synthesized with the following sequences: STAT3 siRNA_1, 5′‐UGAUUCUUCGUAGAUUGUGCU‐3′; STAT3 siRNA_2, 5′‐ACAUUCUUGGGAUUGUUGGUC‐3′; STAT1 siRNA_1, 5′‐AUUAUCCUGAAGAUUACGCUU‐3′; and STAT1 siRNA_2, 5′‐UCAUACUGUCGAAUUCUACAG‐3′. Wild‐type SCD1 plasmids, Myc‐tagged SCD1 point mutants (D44A, D49A, K337R, and K341R), and the corresponding empty vector were also purchased from BPV Biotechnology Co., Ltd. (China). Transient transfection of plasmids or siRNA duplexes was carried out using Lipofectamine 2000 (Invitrogen, USA) according to the manufacturer's instructions. Knockdown and overexpression efficiencies were assessed by western blotting 48 h after transfection.

### 5.6 Data‐independent Acquisition (DIA) Quantitative Proteomics

Quantitative proteomic analysis was performed using ultra‐fast data‐independent acquisition (DIA)‐based LC–MS/MS with technical support from Metware Biotechnology Co., Ltd. (Wuhan, China). Briefly, 100 µg of protein was solubilized in 8 M urea, reduced with 5 mM dithiothreitol at 37°C for 45 min, and alkylated with 11 mM iodoacetamide at room temperature in the dark. Samples were digested overnight at 37°C with trypsin (Promega, V5280), acidified, desalted using C18 cartridges (Millipore), and quantified with a Pierce Quantitative Peptide Assay Kit (Thermo Fisher Scientific). Peptides were separated on a Vanquish Neo UHPLC system (Thermo Fisher Scientific) using a trap‐and‐elute setup with a PepMap Neo trap column and an Easy‐Spray PepMap Neo analytical column. MS analysis was performed on an Orbitrap Astral high‐resolution mass spectrometer (Thermo Fisher Scientific) in positive‐ion DIA mode. Raw data were analyzed using DIA‐NN (v1.8.1) against the UniProt human proteome database, with an FDR of <1% at both precursor and protein levels.

### 5.7 Untargeted Lipidomic Analysis

Untargeted lipidomic profiling was conducted by UHPLC–MS/MS with technical support from Majorbio Biotechnology Co., Ltd. (Shanghai, China). Lipids were extracted using a methyl tert‐butyl ether (MTBE)‐based protocol. Briefly, samples were homogenized in methanol/water and MTBE, followed by ultrasonication and phase separation. The organic phase was collected, dried under nitrogen, and reconstituted in isopropanol/acetonitrile. Pooled quality control samples were analyzed at regular intervals to assess system stability. Lipid separation was performed on an Accucore C30 column coupled to a Q Exactive HF‐X mass spectrometer operated in both positive‐ and negative‐ion modes. Raw data were processed and lipids were annotated using LipidSearch software with a mass tolerance of <10 ppm.

### 5.8 RNA Extraction and Quantitative qRT‐PCR Analysis

Total RNA was isolated from cultured cells using a commercial RNA extraction kit (Omega, R6934‐01). RNA concentration was determined, and equal amounts of RNA were reverse transcribed into complementary DNA (cDNA) using PrimeScript RT Master Mix (Thermo Fisher Scientific, USA). Quantitative real‐time PCR (qRT‐PCR) was carried out to assess target gene expression, with GAPDH as the internal control. Relative expression levels were calculated using the 2^−ΔΔCt method. Primer sequences for additional genes are provided in Table S5.

### 5.9 Western Bot Analysis

Total protein was extracted from cultured cells using radioimmunoprecipitation assay (RIPA) buffer supplemented with protease inhibitor cocktail (Solarbio, China). Protein concentration was measured using a bicinchoninic acid (BCA) protein assay kit (Beyotime, China). Equal amounts of protein were separated by 6%–12% SDS–polyacrylamide gel electrophoresis and transferred onto polyvinylidene difluoride (PVDF) membranes (Millipore, USA). Membranes were blocked with 5% non‐fat milk for 1 h at room temperature and incubated with the indicated primary antibodies at 4°C overnight. After incubation with HRP‐conjugated secondary antibodies for 1 h at room temperature, signals were detected using a chemiluminescent substrate (BL520A, Biosharp, China) on a chemiluminescence imaging system (Tonon 5200, China). Band intensities were quantified using ImageJ software. The antibodies used in this study are listed in Table S4.

### 5.10 Co‐immunoprecipitation (Co‐IP) Assay

Co‐immunoprecipitation (Co‐IP) was carried out using the Pierce Classic Magnetic IP/Co‐IP Kit (PB201, Vazyme, China) according to the manufacturer's instructions. Proteins were extracted in lysis buffer supplemented with protease inhibitor cocktail (Solarbio, China). Lysates were incubated with the indicated antibodies at 4°C overnight to form immune complexes, followed by incubation with magnetic beads for 1 h at room temperature. After three washes with lysis buffer, bound proteins were eluted with 1× SDS loading buffer (P0015, Beyotime, China) and analyzed by western blotting.

### 5.11 Silver Staining and LC‐MS/MS Analysis

Proteins obtained from co‐immunoprecipitation assays were separated by SDS–PAGE and visualized using a rapid silver staining kit (Beyotime, China) according to the manufacturer's instructions. Bands of interest were excised, destained, dehydrated, reduced with dithiothreitol, alkylated with iodoacetamide, and digested overnight with trypsin at 37°C. Peptides were then extracted, vacuum‐dried, desalted, and reconstituted in 0.1% formic acid. Peptide separation was performed on an EASY‐nLC 1200 system using a linear acetonitrile gradient, followed by LC–MS/MS analysis on a Q Exactive HF‐X mass spectrometer operated in data‐dependent acquisition mode. All analyses were carried out by Nanjing BPV Biotechnology Co., Ltd. (China). Full MS scans were acquired over an m/z range of 350–1500 at a resolution of 60,000, and MS/MS spectra were collected at a resolution of 15,000. Raw data were processed using Proteome Discoverer (v2.4) and searched against the UniProt human protein database with trypsin specificity and up to two missed cleavages allowed.

### 5.12 Immunofluorescence

Cells were fixed in 4% paraformaldehyde for 15 min at room temperature and permeabilized with 0.5% Triton X‐100 for 20 min. After blocking with 5% bovine serum albumin (BSA) for 1 h, cells were incubated overnight at 4°C with primary antibodies against PDIA6 (Proteintech, 18233‐1‐AP; 1:1000) and SCD1 (Proteintech, 28678‐1‐AP; 1:1000). After washing, cells were incubated with fluorophore‐conjugated secondary antibodies (Huabio; 1:500) for 1 h at room temperature. Nuclei were counterstained with DAPI (Solarbio, China) for 15 min in the dark. Images were captured from five randomly selected fields using a fluorescence microscope (Zeiss, Germany), and signal intensity was quantified with ImageJ software.

### 5.13 Immunohistochemistry

Tissue microarrays were constructed using 96 paired gastric cancer specimens and matched adjacent non‐tumorous tissues (Fresco Biological Technology Co., Ltd., Nanjing, China). Immunohistochemical staining was independently evaluated by two experienced pathologists in a blinded manner. The proportion of positively stained cells and staining intensity were scored separately, and the final immunoreactivity score was calculated as the product of these two parameters. The proportion of positive cells was scored as 0 (1%–5%), 1 (6%–25%), 2 (26%–50%), 3 (51%–75%), and 4 (76%–100%). Staining intensity was scored as 0 (negative), 1 (weak), 2 (moderate), or 3 (strong). The final score was defined as the mean of the two observers’ scores and categorized as low (0–3), moderate (4–6), or high (7–12) expression.

Tumor tissues from xenograft models were fixed, paraffin‐embedded, and sectioned at 4 µm thickness. After deparaffinization and rehydration, sections were treated with 3% hydrogen peroxide to quench endogenous peroxidase activity. Antigen retrieval was performed in citrate buffer (pH 6.0), followed by blocking with normal goat serum to reduce nonspecific binding. Sections were then incubated with the indicated primary antibodies overnight at 4°C. Immunoreactivity was visualized using 3,3′‐diaminobenzidine (DAB), followed by hematoxylin counterstaining. Images were acquired using a light microscope, and positively stained tumor cells were quantified.

### 5.14 Chromatin Immunoprecipitation (ChIP) Assay

Chromatin immunoprecipitation (ChIP) assays were carried out using a commercial ChIP kit (Beyotime, P2080S) according to the manufacturer's instructions. Briefly, MKN45 cells cultured in 10 cm dishes were harvested, lysed, and sonicated to fragment chromatin (10 s per cycle for 3–4 cycles at 30% amplitude and 50 W). Sheared chromatin was incubated overnight at 4°C with 1 µg of the indicated antibody and Protein A/G magnetic beads pre‐blocked with salmon sperm DNA. After extensive washing, DNA–protein complexes were eluted and treated with proteinase K to reverse crosslinks. Immunoprecipitated DNA was purified and analyzed by quantitative real‐time PCR and agarose gel electrophoresis. Primer sequences and antibodies used in the ChIP assays are provided in Tables S4 and S5.

### 5.15 Double Luciferase Reporter Gene Experiment

Endotoxin‐free plasmids, including pGL4.20‐PDIA6 promoter (WT), pGL4.20‐PDIA6 promoter (Mut), pcDNA3.1, and pcDNA3.1‐STAT3, were prepared using an endotoxin‐free plasmid midiprep kit (Tiangen, China) according to the manufacturer's instructions. HEK293T cells were seeded in 24‐well plates and transfected at approximately 80% confluence using Exfect 2000 Transfection Reagent (Vazyme, China) according to the indicated grouping. Cells were harvested 24 h after transfection, lysed, and subjected to luciferase measurement using the Dual‐Luciferase Reporter Assay Kit (Vazyme, China). Firefly and Renilla luciferase activities were sequentially measured on a GloMax 20/20 luminometer (Promega). Firefly luciferase activity was normalized to Renilla luciferase activity, and normalized values were analyzed using GraphPad Prism.

### 5.16 5‐Ethynyl‐2′‐deoxyuridine (EdU) Assay

Cell proliferation was assessed using an EdU incorporation assay kit (C0078L, Beyotime, China). Cells were incubated with 100 µM EdU at 37°C for 2 h, followed by fixation with 4% paraformaldehyde and permeabilization with 0.5% Triton X‐100. Cells were then reacted with Click Additive Solution for 30 min according to the manufacturer's instructions. Nuclei were counterstained with Hoechst 33342 (C1022, Beyotime, China) at a final concentration of 10 µg/mL for 10 min at room temperature in the dark. EdU‐positive cells were quantified to evaluate proliferative activity.

### 5.17 Cell Migration Assay

Cell migration was evaluated using Transwell chambers with 8 µm pore‐size membranes (Corning, Cat. No. 3422). Briefly, 2 × 10^4 cells in serum‐free medium were seeded into the upper chamber, and medium supplemented with 20% fetal bovine serum was placed in the lower chamber as a chemoattractant. After incubation at 37°C for 24–48 h, cells remaining on the upper membrane surface were removed. Cells that had migrated to the lower surface were fixed with 4% paraformaldehyde, stained with 0.1% crystal violet, and quantified in at least five randomly selected fields under a light microscope.

### 5.18 Preparation of Conditioned Medium From NFs and CAFs

Conditioned medium (CM) was collected from NFs and CAFs at approximately 80% confluence. After removal of the culture medium, cells were washed three times with sterile phosphate‐buffered saline (PBS) and incubated in serum‐free or 1% fetal bovine serum (FBS)‐supplemented DMEM for 48 h. Supernatants were then harvested, centrifuged at 300 × g for 5 min to remove cellular debris, and passed through 0.22 µm filters. The resulting CM was stored at −80°C until use in subsequent functional assays.

### 5.19 Enzyme‐Linked Immunosorbent Assay (ELISA)

CXCL12 levels in cell culture supernatants were quantified using a human CXCL12 ELISA kit (RK00266, Abclonal, China) according to the manufacturer's instructions. Absorbance was measured at 450 nm on a microplate reader (BioTek, USA).

### 5.20 Detection of Lipid Droplets by Fluorescence Microscopy

Intracellular lipid droplets were detected using a BODIPY 493/503 staining kit (C2053S, Beyotime, China). Cells were seeded in 6‐well plates, treated as indicated, and washed with pre‐warmed phosphate‐buffered saline (PBS). Cells were then incubated with BODIPY 493/503 staining solution for 10–20 min at room temperature in the dark. After washing, nuclei were counterstained with Hoechst 33342 (10 µg/mL) for 10 min at room temperature in the dark. Images were captured immediately using a fluorescence microscope under identical acquisition settings. Lipid droplet accumulation was quantified as the mean BODIPY 493/503 fluorescence intensity per cell using ImageJ, with at least 100 cells analyzed per independent experiment.

### 5.21 Lipid Peroxidation Detection Assay

Lipid peroxidation was detected using a C11‐BODIPY (581/591) fluorescent probe kit (S0043S, Beyotime, China). Cells were seeded in 6‐well plates, treated as indicated, washed with pre‐warmed phosphate‐buffered saline (PBS), and incubated with C11‐BODIPY (581/591) working solution (2–5 µM) at 37°C for 20–30 min in the dark. After washing, nuclei were counterstained with Hoechst 33342 (10 µg/mL) for 10 min at room temperature in the dark. Cells were then washed again and immediately imaged by fluorescence microscopy under identical acquisition settings. Oxidized C11‐BODIPY was detected in the green channel (FITC, ∼510–550 nm), whereas the reduced probe was detected in the red channel (PE/TRITC, ∼580–620 nm). Lipid peroxidation levels were quantified as the shift from red to green fluorescence or as the green/red fluorescence ratio.

### 5.22 Transmission Electron Microscopy

GC cells were washed with phosphate‐buffered saline (PBS) and pre‐fixed in 2.5% glutaraldehyde (G1102, Servicebio, China). Cells were gently scraped, pelleted by centrifugation at ≤3000 rpm for approximately 2 min, and resuspended in fresh 2.5% glutaraldehyde for fixation. Ultrastructural analysis was then performed using a transmission electron microscope (HT7700, Hitachi, Japan).

### 5.23 Molecular Docking Analysis

The 3D structures of PDIA6 (UniProt: Q15084) and SCD1 (UniProt: O00767) were retrieved from the RCSB Protein Data Bank. Structural modeling was performed using AlphaFold 3 (version 2024‐05‐01). FASTA sequences of PDIA6 and SCD1 were submitted using default settings, without homologous templates or structural restraints, so that all models were generated solely by the deep learning‐based prediction framework. SCD1 mutant sequences were generated by introducing the indicated point substitutions into the wild‐type sequence and were independently modeled using the same AlphaFold 3 workflow. Model quality was evaluated using the built‐in AlphaFold confidence metrics. The predicted template modeling (pTM) scores were 0.82 for PDIA6 and 0.79 for wild‐type SCD1, both above the recommended threshold for reliable global folding (pTM > 0.70). For each protein, the model with the highest pTM score was selected for downstream analyses. Protein–protein docking was carried out using HDOCK with a grid spacing of 1.200 and an angular step size of 15.000 to refine sampling of candidate interaction interfaces. Docking poses were ranked by docking score, and the top‐ranked complex was visualized and analyzed in PyMOL to define binding conformations and residue‐level interactions.

### 5.24 Molecular Dynamic Simulations

Molecular dynamics simulations were carried out using GROMACS 2022.3 [63, 64]. Small‐molecule ligands were parameterized with the general AMBER force field (GAFF) in AmberTools22. Hydrogen atoms were added, and restrained electrostatic potential (RESP) charges were calculated using Gaussian 16 W, after which the resulting parameters were incorporated into the system topology. The AMBER99SB‐ILDN force field was used for the protein, and the system was solvated with the TIP3P water model. Na^+ ions were added to neutralize the system. Energy minimization was performed using the steepest descent algorithm, followed by 100 ps equilibration under both NVT and NPT ensembles at 300 K and 1 bar with a coupling constant of 0.1 ps. Production runs were then conducted for 100 ns with a time step of 2 fs. Trajectories were analyzed using built‐in GROMACS tools to calculate the root mean square deviation (RMSD), root mean square fluctuation (RMSF), and radius of gyration. Binding free energies were further estimated by MM/GBSA, together with free energy landscape analysis.

### 5.25 Cycloheximide (CHX) Chase and Inhibitor Treatments

For CHX chase assays, PDIA6‐knockdown MKN45 cells and PDIA6‐overexpressing AGS cells were treated with cycloheximide (CHX, 20 µM) to block de novo protein synthesis. Cells were collected at the indicated time points (0, 2, 4, 6, 8, and 12 h), and protein lysates were subjected to western blot analysis to evaluate the degradation kinetics of SCD1.

For inhibitor treatment assays, PDIA6‐knockdown MKN45 cells and PDIA6‐overexpressing AGS cells were treated with the autophagy‐lysosome inhibitor chloroquine (CQ, 20 µM) for 12 h or proteasome inhibitor MG132 (20 µM) for 6 h before protein extraction. For the combined treatment assay, cells were treated with CHX (20 µM) for 12 h, with or without MG132 (20 µM) added during the final 6 h, followed by western blot analysis of SCD1 protein levels.

### 5.26 In Vivo Ubiquitination Assays

In vivo ubiquitination assays were performed in HEK‐293T cells co‐transfected with HA‐tagged ubiquitin, Myc‐tagged SCD1, and the indicated PDIA6 overexpression or knockdown constructs. At 48 h after transfection, cells were pretreated with MG132 (20 µM) for 6 h before harvest to block proteasome‐dependent degradation and allow accumulation of polyubiquitinated SCD1. Cell lysates were subjected to immunoprecipitation using anti‐Myc agarose beads to enrich Myc‐SCD1, followed by western blotting to assess its ubiquitination status.

### 5.27 In Vivo Experiments

All animal procedures were approved by the Animal Ethics Committee of Northern Jiangsu People's Hospital (approval No. 2024ky192) and conducted in accordance with institutional guidelines. BALB/c nude mice (4–6 weeks old) were maintained under specific pathogen‐free conditions at 24°C on a 12 h light/dark cycle and randomly assigned to the indicated experimental groups.

For subcutaneous xenograft experiments, MKN45 cells stably expressing control shRNA or shPDIA6 were resuspended in PBS and subcutaneously injected into the flanks of BALB/c nude mice (5 × 10^6^ cells per mouse). Tumor dimensions were measured every 3 days, and tumor volume was calculated using the formula V  =  (*a* × *b* ^2^)/2, where *a* and *b* represent the longest and shortest tumor diameters, respectively. At the endpoint, mice were euthanized, and tumors were excised, weighed, and subjected to hematoxylin and eosin staining, histopathological evaluation, and immunohistochemical analysis.

For liver metastasis experiments, 1 × 10^6^ MKN45 cells suspended in 50 µL PBS were injected into the spleen to establish splenic injection‐derived hepatic metastasis models without splenectomy. Hepatic metastatic burden was evaluated 4–5 weeks later by fluorescence imaging, gross inspection, and histological examination.

For pharmacological intervention studies, mice bearing either subcutaneous xenografts or splenic injection‐derived liver metastases were treated with the SCD1 inhibitor CAY10566 (10 mg/kg) or vehicle control (0.01% DMSO) according to the indicated dosing schedules. Tumor growth, metastatic progression, and general condition were monitored throughout the treatment period, and no overt systemic toxicity was observed.

### 5.28 Statistical Analysis

Statistical analyses were performed using R software (version 4.1.3) and GraphPad Prism (version 10.1.2). Data normality was assessed using the Shapiro–Wilk test, and homogeneity of variance was evaluated using Bartlett's test. Quantitative data are presented as the mean ± standard deviation (SD). Comparisons between two independent groups were performed using unpaired Student's t‐tests, whereas comparisons among multiple groups were conducted using one‐way analysis of variance (ANOVA). Two‐way ANOVA was applied for experiments involving two independent variables. Nonparametric data were analyzed using the Wilcoxon test. Pearson correlation analysis was used to assess associations between variables. Survival differences were evaluated using Kaplan–Meier analysis with the log‐rank test. A two‐sided *p* value < 0.05 was considered statistically significant. Statistical significance was denoted as *p < 0.05, **p < 0.01, and *p < 0.001.

## Funding

This work was supported by the National Natural Science Foundation of China (NO. 82373014).

## Conflicts of Interest

The authors declare no conflicts of interest.

## Author Contributions

Zhen Tian and Yifan Cheng contributed equally to this work. Sen Wang and Daorong Wang conceived and supervised the study. Zhen Tian and Yifan Cheng performed the experiments, prepared the figures, and drafted the manuscript. Clinical specimen and data collection were carried out by Jiajie Zhou, Ruiqi Li, Shuai Zhao, Ben Li, Zijie Xu, Mengli Zi, Yayan Fu, Chenkai Zhang, and Qiannan Sun. Bioinformatic analyses were performed by Zhen Tian and Ben Li. All authors reviewed and approved the final manuscript. All authors agree to submit the article for publication.

## Supporting information




**Supporting File 1**: advs75923‐sup‐0001‐FigureS1‐S8.docx.

**Supporting File 2**: advs75923‐sup‐0002‐TableS1‐S5.docx.

**Supporting File 3**: advs75923‐sup‐0003‐ClusterCOX.xlsx.

## Data Availability

The RNA sequencing datasets generated and/or analyzed in this study are available in the Gene Expression Omnibus (GEO) repository under accession numbers GSE163558, GSE56807, and GSE183904, and are accessible upon publication. Additional data supporting the findings of this study are available from the corresponding authors upon reasonable request.
