# Supplementary material for: PDIA6–SCD1 Axis Rewires Lipid Metabolism to Drive Gastric Cancer Progression
Source: Adv Sci (Weinh). 2026 Jun 3;13(48):e75923. doi: 10.1002/advs.75923 (PMC13336654; doi:10.1002/advs.75923)
Supplement: Supplementary file 1 [file ADVS-13-e75923-s001.docx]

**PDIA6–SCD1 Axis Rewires Lipid Metabolism to Drive Gastric Cancer Progression**

**Authors**

Zhen Tian ^1^, Yifan Cheng ^1^, Jiajie Zhou ^1^, Ruiqi Li ^1^, Shuai Zhao ^1^, Ben Li ^1^, Zijie Xu ^1^, Mengli Zi ^1^, Yayan Fu ^2^, Chenkai Zhang ^2^, Qiannan Sun ^3^, Shantanu Baral ^4^, Sen Wang ^5,*^ and Daorong Wang ^1,2,4,*^

* **Corresponding Authors**

Sen Wang, E-mail: [wangsen1992@hotmail.com](mailto:wangsen1992@hotmail.com);

Daorong Wang, E-mail: [wdaorong666@sina.com](mailto:wdaorong666@sina.com).

Zhen Tian and Yifan Cheng contributed equally as co-first authors.

**Affiliations**

1 Northern Jiangsu People's Hospital, Clinical Teaching Hospital of Medical School, Nanjing University, Yangzhou, China

2 Northern Jiangsu People's Hospital Affiliated to Yangzhou University

3 Yangzhou Key Laboratory of Basic and Clinical Transformation of Digestive and Metabolic Diseases, Yangzhou, China

4 Department of General Surgery, Northern Jiangsu People's Hospital, Yangzhou, China

5 Department of General Surgery, The First Affiliated Hospital of Nanjing Medical University, Nanjing, China

**Supplementary Figures and Legends:**

**Figure S1**. Survival correlation of PDIA6 in GC. A) Dot plot of marker gene expression across major cell populations in GC scRNA-seq data (GSE163558). B) Kaplan–Meier survival analysis of other ssGSEA-defined gene clusters in the TCGA-STAD cohort. C) Kaplan–Meier survival analysis of individual genes within cluster26 in the GSE84426 cohort. D) Kaplan–Meier survival analysis of individual genes within cluster26 in the TCGA-STAD cohort. E) scRNA-seq analysis showing PDIA6 expression across major cell types.

**Figure S2.** Quality control and global profiling of DIA-based proteomic analysis. A) Distribution of protein abundance across all samples, indicating comparable signal intensity ranges and overall consistency between groups. B) Principal component analysis (PCA) of proteomic profiles, showing clear separation between control and PDIA6-knockdown samples. C) Pearson correlation heatmap demonstrating high intra-group reproducibility and strong consistency among biological replicates. D) K-means clustering of differentially expressed proteins, revealing distinct proteomic patterns associated with PDIA6 depletion.

**Figure S3.** Quality control and global profiling of untargeted lipidomic analysis. A) Sample correlation heatmap showing overall similarity among lipidomic samples. B) Principal component analysis (PCA) of lipidomic data, showing tight clustering of quality control (QC) samples and indicating good reproducibility and analytical stability. C) Distribution of identified lipid species across major lipid classes and subclasses. D) Bar plot showing the distribution of lipid species according to fatty acyl chain length. E) Pie chart showing the distribution of lipid species according to fatty acyl chain saturation. F) Volcano plot of differential lipid species between groups, with log₂(fold change) plotted against −log₁₀(P value); dot size indicates the VIP score. G) Hierarchical clustering heatmap of differentially abundant lipid species across samples. H) Scatter plot of differentially abundant lipid species, with log₂(fold change) on the x-axis and lipid subclass on the y-axis. I) Bar plot showing the total abundance of lipid subclasses across experimental groups. J) KEGG pathway enrichment analysis of differential lipid species, with differential abundance (DA) scores indicating overall pathway-level changes.

**Figure S4.** Structural interactions between PDIA6 and SCD1. A) Hydrogen bond interactions between PDIA6 and SCD1, with distances (Å) between donor and acceptor residues indicated. B) Salt bridge interactions between PDIA6 and SCD1, with distances (Å) between the residues involved indicated. C) Domain architecture of PDIA6. PDIA6 contains two thioredoxin-like catalytic domains (a and a′), each harboring conserved redox-active cysteine residues, followed by a C-terminal non-catalytic thioredoxin-like domain. D) Domain architecture of SCD1. SCD1 belongs to the membrane-bound fatty acid desaturase (Membrane-FADS-like) superfamily and contains conserved di-iron-binding motifs required for its Δ9-desaturase activity. E) Comparison of molecular dynamics simulation parameters between the PDIA6–SCD1 complex and the SCD1 monomer.

**Figure S5.** Additional validation of invasive phenotypes and upstream transcriptional regulation of PDIA6. A,B) Representative images and quantification of Transwell migration assays. C) Relative PDIA6 mRNA expression in AGS and MKN45 cells after siRNA-mediated STAT1 knockdown, as determined by qRT–PCR. D) Relative PDIA6 mRNA expression in AGS and MKN45 cells following treatment with the NF-κB inhibitor BAY117082 at the indicated concentrations, as measured by qRT–PCR. Data are presented as mean ± SD, and statistical significance is indicated.

**Figure S6.** Pharmacological inhibition and metabolic rescue experiments validate the requirement of SCD1 activity in PDIA6-driven phenotypes. A) Representative images and quantification of BODIPY 493/503 staining in the indicated groups: OE‑Ctrl + vehicle, OE‑Ctrl + CAY10566, OE‑PDIA6 + vehicle, OE‑PDIA6 + CAY10566, and OE‑PDIA6 + CAY10566 + oleic acid (OA). B) Representative images and quantification of BODIPY 581/591 C11 staining. C) Representative images and quantification of EdU incorporation assays. D) Flow cytometric analysis of cell-cycle distribution, with quantification of the percentages of cells in G1, S, and G2 phases. E) Representative images and quantification of Transwell migration assays. AGS cells stably expressing empty vector (OE‑Ctrl) or PDIA6 (OE‑PDIA6) were treated as follows: vehicle (DMSO/BSA); CAY10566 (1 μM, 24 h); or CAY10566 pretreatment for 4–6 h followed by OA supplementation (10 μM) without washout for an additional 24 h. Data are presented as mean ± SD. **p* < 0.05, ***p* < 0.01, ****p* < 0.001; ns, not significant.

**Figure S7.** Metabolic rescue experiments validate the functional contribution of downstream lipid metabolic output to phenotypes induced by PDIA6 knockdown. A) Representative images and quantification of BODIPY 493/503 staining in the indicated groups: sh-Ctrl + BSA, sh-Ctrl + oleic acid (OA), sh-PDIA6 + BSA, and sh-PDIA6 + OA. B) Representative images and quantification of BODIPY 581/591 C11 staining. C) Representative images and quantification of EdU incorporation assays. D) Flow cytometric analysis of cell-cycle distribution and quantification of the percentage of cells in G1, S, and G2 phases. E) Representative images and quantification of Transwell migration assays. MKN45 cells stably expressing control shRNA (sh-Ctrl) or PDIA6 shRNA (sh-PDIA6) were treated with vehicle (BSA) or OA (10 μM) for 24 h. Data are presented as mean ± SD. **p* < 0.05, ***p* < 0.01, ****p* < 0.001; ns, not significant.

**Figure S8**. Characterization of CAF-associated upstream cues linked to PDIA6 expression. A) Isolation and characterization of CAFs and paired normal fibroblasts (NFs) from fresh gastric cancer specimens. B) Correlation analysis between CAF-enriched secreted factors and PDIA6 expression. C) Dot plot showing marker gene expression across major cell populations in GC single-cell RNA-seq data (GSE183904). D) Correlation analysis between PDIA6 and CXCR4, JAK1, and JAK2 expression in the TCGA-STAD cohort. E) Pan-cancer expression profile of PDIA6 based on TCGA data. Data are presented as mean ± SD. **p* < 0.05, ***p* < 0.01, *****p* < 0.001; *****p* < 0.001; ns, not significant.
