# Supplementary material for: PDIA6–SCD1 Axis Rewires Lipid Metabolism to Drive Gastric Cancer Progression
Source: Adv Sci (Weinh). 2026 Jun 3;13(48):e75923. doi: 10.1002/advs.75923 (PMC13336654; doi:10.1002/advs.75923)
Supplement: Supplementary file 2 [file ADVS-13-e75923-s003.docx]

**Supplementary Table S1. Univariate and multivariate Cox regression analysis of risk factors associated with overall survival.**

| **Clinicopathological variables** | **Univariate analysis** | | |  | **Multivariate analysis** | | |
| --- | --- | --- | --- | --- | --- | --- | --- |
|  | **HR** | **95% CI** | **P Value** |  | **HR** | **95% CI** | **P Value** |
| PDIA6 expression |  |  |  |  |  |  |  |
| High | 5.412 | 4.154-6.302 | <0.001 |  | 4.021 | 3.112-4.881 | <0.001 |
| Low | 1 | Reference | - |  | 1 | Reference | - |
| Gender |  |  |  |  |  |  |  |
| Male | 1.528 | 0.713-2.473 | 0.238 |  | - | - | - |
| Female | 1 | Reference | - |  | - | - | - |
| Age |  |  |  |  |  |  |  |
| ≥70 | 1.758 | 0.846-2.605 | 0.119 |  | - | - | - |
| <70 | 1 | Reference | - |  | - | - | - |
| Differentiation |  |  |  |  |  |  |  |
| Poor | 2.755 | 1.922-3.558 | 0.011 |  | 1.964 | 1.115-2.723 | 0.021 |
| Well | 1 | Reference | - |  | 1 | Reference | - |
| Perineural invasion |  |  |  |  |  |  |  |
| Positive | 2.106 | 1.222-2.905 | 0.021 |  | 1.616 | 0.702-2.505 | 0.106 |
| Negative | 1 | Reference | - |  | 1 | Reference | - |
| CEA |  |  |  |  |  |  |  |
| ≥10 ng/ml | 2.281 | 1.008-3.154 | 0.038 |  | 1.752 | 1.335-2.889 | 0.062 |
| <10 ng/ml | 1 | Reference | - |  | 1 | Reference | - |
| TNM stage |  |  |  |  |  |  |  |
| III/IV | 4.022 | 2.899-5.155 | <0.001 |  | 2.998 | 2.103-3.923 | <0.001 |
| I/II | 1 | Reference | - |  | 1 | Reference | - |

**Supplementary Table S2. Univariate and multivariate Cox regression analysis of risk factors associated with disease-free survival.**

| **Clinicopathological variables** | **Univariate analysis** | | |  | **Multivariate analysis** | | |
| --- | --- | --- | --- | --- | --- | --- | --- |
|  | **HR** | **95% CI** | **P Value** |  | **HR** | **95% CI** | **P Value** |
| PDIA6 expression |  |  |  |  |  |  |  |
| High | 4.051 | 2.771-6.805 | <0.001 |  | 3.631 | 1.955-5.168 | 0.006 |
| Low | 1 | Reference | - |  | 1 | Reference | - |
| Gender |  |  |  |  |  |  |  |
| Male | 0.844 | 0.651-1.086 | 0.297 |  |  |  |  |
| Female | 1 | Reference | - |  |  |  |  |
| Age |  |  |  |  |  |  |  |
| ≥70 | 1.374 | 0.782-2.335 | 0.259 |  |  |  |  |
| <70 | 1 | Reference | - |  | 1 | Reference | - |
| Differentiation |  |  |  |  |  |  |  |
| Poor | 2.601 | 1.054-4.496 | 0.010 |  | 1.985 | 1.082-3.416 | 0.032 |
| Well | 1 | Reference | - |  | 1 | Reference | - |
| Perineural invasion |  |  |  |  |  |  |  |
| Positive | 1.515 | 0.781-2.527 | 0.142 |  |  |  |  |
| Negative | 1 | Reference | - |  |  |  |  |
| CEA |  |  |  |  |  |  |  |
| ≥10 ng/ml | 1.922 | 1.045-2.758 | 0.042 |  | 1.426 | 0.961-2.104 | 0.084 |
| <10 ng/ml | 1 | Reference | - |  |  |  |  |
| TNM stage |  |  |  |  |  |  |  |
| III/IV | 2.295 | 1.392-4.101 | 0.013 |  | 2.038 | 1.156-3.741 | 0.026 |
| I/II | 1 | Reference | - |  | 1 | Reference | - |

**Supplementary Table S3. Point Mutation Sequences of SCD1**

| **Mutation** | **Sequence** |
| --- | --- |
| **WT** | *MPAHLLQDDISSSYTTTTTITAPPSRVLQNGGDKLETMPLYLE****D****DIRP****D****IKDDIYDPTYKDKEGPSPKVEYVWRNIILMSLLHLGALYGITLIPTCKFYTWLWGVFYYFVSALGITAGAHRLWSHRSYKARLPLRLFLIIANTMAFQNDVYEWARDHRAHHKFSETHADPHNSRRGFFFSHVGWLLVRKHPAVKEKGSTLDLSDLEAEKLVMFQRRYYKPGLLMMCFILPTLVPWYFWGETFQNSVFVATFLRYAVVLNATWLVNSAAHLFGYRPYDKNISPRENILVSLGAVGEGFHNYHHSFPYDYSASEYRWHINFTTFFIDCMAALGLAYDR****K****KVS****K****AAILARIKRTGDGNYKSG* |
| **D44A** | *MPAHLLQDDISSSYTTTTTITAPPSRVLQNGGDKLETMPLYLE****A****DIRP****D****IKDDIYDPTYKDKEGPSPKVEYVWRNIILMSLLHLGALYGITLIPTCKFYTWLWGVFYYFVSALGITAGAHRLWSHRSYKARLPLRLFLIIANTMAFQNDVYEWARDHRAHHKFSETHADPHNSRRGFFFSHVGWLLVRKHPAVKEKGSTLDLSDLEAEKLVMFQRRYYKPGLLMMCFILPTLVPWYFWGETFQNSVFVATFLRYAVVLNATWLVNSAAHLFGYRPYDKNISPRENILVSLGAVGEGFHNYHHSFPYDYSASEYRWHINFTTFFIDCMAALGLAYDR****K****KVS****K****AAILARIKRTGDGNYKSG* |
| **D49A** | *MPAHLLQDDISSSYTTTTTITAPPSRVLQNGGDKLETMPLYLE****D****DIRP****A****IKDDIYDPTYKDKEGPSPKVEYVWRNIILMSLLHLGALYGITLIPTCKFYTWLWGVFYYFVSALGITAGAHRLWSHRSYKARLPLRLFLIIANTMAFQNDVYEWARDHRAHHKFSETHADPHNSRRGFFFSHVGWLLVRKHPAVKEKGSTLDLSDLEAEKLVMFQRRYYKPGLLMMCFILPTLVPWYFWGETFQNSVFVATFLRYAVVLNATWLVNSAAHLFGYRPYDKNISPRENILVSLGAVGEGFHNYHHSFPYDYSASEYRWHINFTTFFIDCMAALGLAYDR****K****KVS****K****AAILARIKRTGDGNYKSG* |
| **K337R** | *MPAHLLQDDISSSYTTTTTITAPPSRVLQNGGDKLETMPLYLE****D****DIRP****D****IKDDIYDPTYKDKEGPSPKVEYVWRNIILMSLLHLGALYGITLIPTCKFYTWLWGVFYYFVSALGITAGAHRLWSHRSYKARLPLRLFLIIANTMAFQNDVYEWARDHRAHHKFSETHADPHNSRRGFFFSHVGWLLVRKHPAVKEKGSTLDLSDLEAEKLVMFQRRYYKPGLLMMCFILPTLVPWYFWGETFQNSVFVATFLRYAVVLNATWLVNSAAHLFGYRPYDKNISPRENILVSLGAVGEGFHNYHHSFPYDYSASEYRWHINFTTFFIDCMAALGLAYDR****R****KVS****K****AAILARIKRTGDGNYKSG* |
| **K341R** | *MPAHLLQDDISSSYTTTTTITAPPSRVLQNGGDKLETMPLYLE****D****DIRP****D****IKDDIYDPTYKDKEGPSPKVEYVWRNIILMSLLHLGALYGITLIPTCKFYTWLWGVFYYFVSALGITAGAHRLWSHRSYKARLPLRLFLIIANTMAFQNDVYEWARDHRAHHKFSETHADPHNSRRGFFFSHVGWLLVRKHPAVKEKGSTLDLSDLEAEKLVMFQRRYYKPGLLMMCFILPTLVPWYFWGETFQNSVFVATFLRYAVVLNATWLVNSAAHLFGYRPYDKNISPRENILVSLGAVGEGFHNYHHSFPYDYSASEYRWHINFTTFFIDCMAALGLAYDR****K****KVS****R****AAILARIKRTGDGNYKSG* |

**Supplementary Table S4. The information of primary antibodies**

| **Antibody** | **Assay** | **Host Species** | **Company** | **Catalog Number** | **Dilution ration** |
| --- | --- | --- | --- | --- | --- |
| GAPDH | WB | Rabbit | Proteintech | 10494-1-AP | 1: 100000 |
| β-Tubulin | WB | Rabbit | Abclonal | A12289 | 1: 10000 |
| PDIA6 | WB | Rabbit | Proteintech | 18233-1-AP | 1: 1000 |
|  | IP |  |  |  | 4 μl per 1 mg of total protein |
|  | IHC |  |  |  | 1:200 |
|  | IF |  |  |  | 1:100 |
| SCD1 | WB | Rabbit | Proteintech | 28678-1-AP | 1:500 |
|  | IHC |  |  |  | 1:200 |
|  | IF |  |  |  | 1:100 |
| SCD1 | IP | Rabbit | Abcam | ab236868 | 5 μl per 1 mg of total protein |
| STAT3 | WB | Rabbit | Proteintech | 10253-2-AP | 1:5000 |
|  | ChIP |  |  |  |  |
| p-STAT3 (Tyr705) | WB | Rabbit | Proteintech | 39595 | 1:1000 |
| E-cadherin | WB | Rabbit | Proteintech | 20874-1-AP | 1:10000 |
| N-cadherin | WB | Rabbit | Proteintech | 22018-1-AP | 1:2000 |
| Vimentin | WB | Rabbit | Proteintech | 10366-1-AP | 1:20000 |
| FAP | WB | Rabbit | Proteintech | 84018-4-RR | 1:1000 |
| α-SMA | WB | Rabbit | Proteintech | 14395-1-AP | 1:2000 |
| CXCL12 | WB | Rabbit | Proteintech | 17402-1-AP | 1:1000 |
| IgG | IP | Rabbit | Proteintech | 10284-1-AP | 2 μl per 1 mg of total protein |
| HRP-conjugated Goat | WB | Rabbit | Abclonal | AS014 | 1:10000 |
| Anti Myc-Tag | WB | Rabbit | Abclonal | AE009 | 1:2000 |
|  | IP |  |  |  | 5 μl per 1 mg of total protein |
| Anti HA-Tag | WB | Rabbit | Abclonal | AE036 | 1:1000 |
|  | IP |  |  |  | 5 μl per 1 mg of total protein |
| Anti Flag-Tag | WB | Rabbit | Abclonal | AE063 | 1:5000 |
|  | IP |  |  |  | 5 μl per 1 mg of total protein |

**Supplementary Table S5. Sequences of oligonucleotides used for real-time quantitative PCR.**

| Primer | Species | Forward/ Reverse | Sequence (5'-3') |
| --- | --- | --- | --- |
| GAPDH | Homos | Forward | TCCAAAATCAAGTGGGGCGA |
|  |  | Reverse | *AAATGAGCCCCAGCCTTCTC* |
| PDIA6 | Homos | Forward | *AAGAAAATGTGGGGGTGGCT* |
|  |  | Reverse | *ATCTTGCGTGCATTGATGGC* |
| PDIA6 ChIP Primer 1 | Homos | Forward | *TGCAGGAAAGGGGTCAGAAA* |
|  |  | Reverse | *GCAGCTCCTCCTGAATGTGA* |
| PDIA6 ChIP Primer 2 | Homos | Forward | *AATCAATGCTGGCATGTGGC* |
|  |  | Reverse | *GTTTGGAGTGGACTCGGACA* |
| SCD1 | Homos | Forward | *TTCCCGACGTGGCTTTTTCT* |
|  |  | Reverse | *AGCCAGGTTTGTAGTACCTCC* |
| STAT3 | Homos | Forward | *GAGCTGCACCTGATCACCTT* |
|  |  | Reverse | *TGGCAAGGAGTGGGTCTCTA* |
